# Microbial metabolites in tumor metabolic reprogramming and immunotherapy: new insights

**DOI:** 10.3389/fcimb.2025.1706040

**Published:** 2025-11-06

**Authors:** Yi Wang, Dan Huang, Rongrong Wang, Jiaojiao Yin, Jianying Pei, Chong Zhang

**Affiliations:** 1 School of Public Health, Gansu University of Chinese Medicine, Lanzhou, Gansu, China; 2 Department of Clinical Laboratory center, Gansu Provincial Maternity and Child-Care Hospital, Lanzhou, Gansu, China

**Keywords:** gut microbiota, microbial metabolites, tumor metabolic reprogramming, immune cell reprogramming, cancer immunotherapy, clinical translation

## Abstract

Gut microbiota and intratumoral microbiota have been recognized as critical regulators of tumor initiation and progression, with their metabolites exerting multifaceted effects on cancer development. This review systematically summarizes the roles and mechanistic classifications of microbial metabolites in tumor metabolic reprogramming, highlighting three principal regulatory layers: direct modulation of metabolic enzyme activity, regulation via signaling pathways, and immunometabolic modulation. Furthermore, it discusses the potential of microbial metabolites in regulating immune cell metabolism and enhancing immunotherapeutic efficacy, alongside summarizing the progress of relevant clinical studies. Finally, the review emphasizes that despite significant advances, current research still faces multiple challenges. Future investigations integrating cutting-edge technologies are essential to accelerate the translation of fundamental findings into clinical applications and to promote the development of metabolite-centered precision therapeutic strategies.

## Graphical Abstract

Schematic illustration of the role of microbial metabolites in tumor metabolism and progression. Gut microbiota and intratumoral bacteria generate metabolites that modulate metabolic reprogramming in tumor cells and tumor-associated immune cells. This reprogramming, in turn, influences tumor progression, including growth, metastasis, and apoptosis, and also affects therapeutic responses such as chemotherapy, immunotherapy, and hormonal therapy. Created in https://BioRender.com.

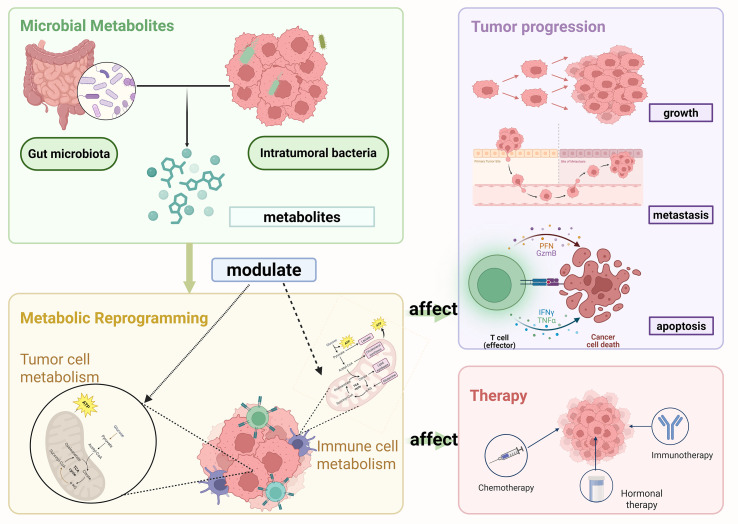

## 1 Introduction

Cancer is now one of the leading causes of death worldwide. A thorough understanding of the biological characteristics of cancer cells is crucial. Douglas and colleagues systematically summarized the ten hallmarks of cancer, including sustained proliferative signaling, evasion of growth suppressors, resistance to cell death, and metabolic reprogramming, among others (Hanahan and Weinberg, 2011). Among these hallmarks, metabolic reprogramming has emerged as a rapidly advancing field and is now recognized as central to understanding how cancer cells adapt to the complex TME. Under stress conditions, including nutrient deprivation, hypoxia, and immunosuppression, cancer cells dynamically reprogram their energy metabolism and nutrient utilization to maintain survival and support continuous proliferation (Li et al., 2025; Wen et al., 2025). Furthermore, immune cells within the tumor microenvironment undergo metabolic reprogramming, and their crosstalk with cancer cells regulates immune evasion and affects the response to immunotherapy (Wang et al., 2025).

Gut microbiota and intratumoral microbiota, as research hotspots, have been shown to be closely associated with tumor development, providing new entry points for studying the biological characteristics of cancer cells. Studies have found that microbes can directly influence cancer cell growth and metabolism by producing various metabolites. These metabolites can regulate tumor metabolic reprogramming, including glucose, amino acid, and lipid metabolism, For example, SCFAs derived from gut microbiota can modulate glucose metabolism in cancer cells by inhibiting histone deacetylase (HDAC) activity, thereby suppressing tumor cell proliferation and inducing differentiation (Hinnebusch et al., 2002). Furthermore, specific microbial metabolites can shape the tumor immune microenvironment, influence immune cell function, and consequently impact immune evasion and therapeutic response. For example, elevated lactate levels impair the activity of CD8^+^ T cells and NK cells, reducing their cytotoxicity and cytokine production, and thereby exacerbating local immune escape (Wang et al., 2023a). These findings suggest that microbial metabolites play a critical role in regulating tumor metabolism and the immune microenvironment and may represent novel strategies or potential targets for improving therapeutic efficacy in cancer.

This review focuses on the role of microbial metabolites in regulating tumor metabolism and modulating antitumor immune responses, viewed through the lens of cancer cell metabolic reprogramming and immunotherapy. By systematically outlining these mechanisms, we underscore their potential influence on tumor initiation, progression, and therapeutic efficacy. Despite the inherent complexity of the microbiota and the diversity of its metabolites, further studies are required to elucidate the underlying molecular pathways and to design strategies that combine metabolic modulation with immunotherapy, thereby providing new opportunities for cancer therapy.

## 2 Microbial metabolites

**Figure 1:**
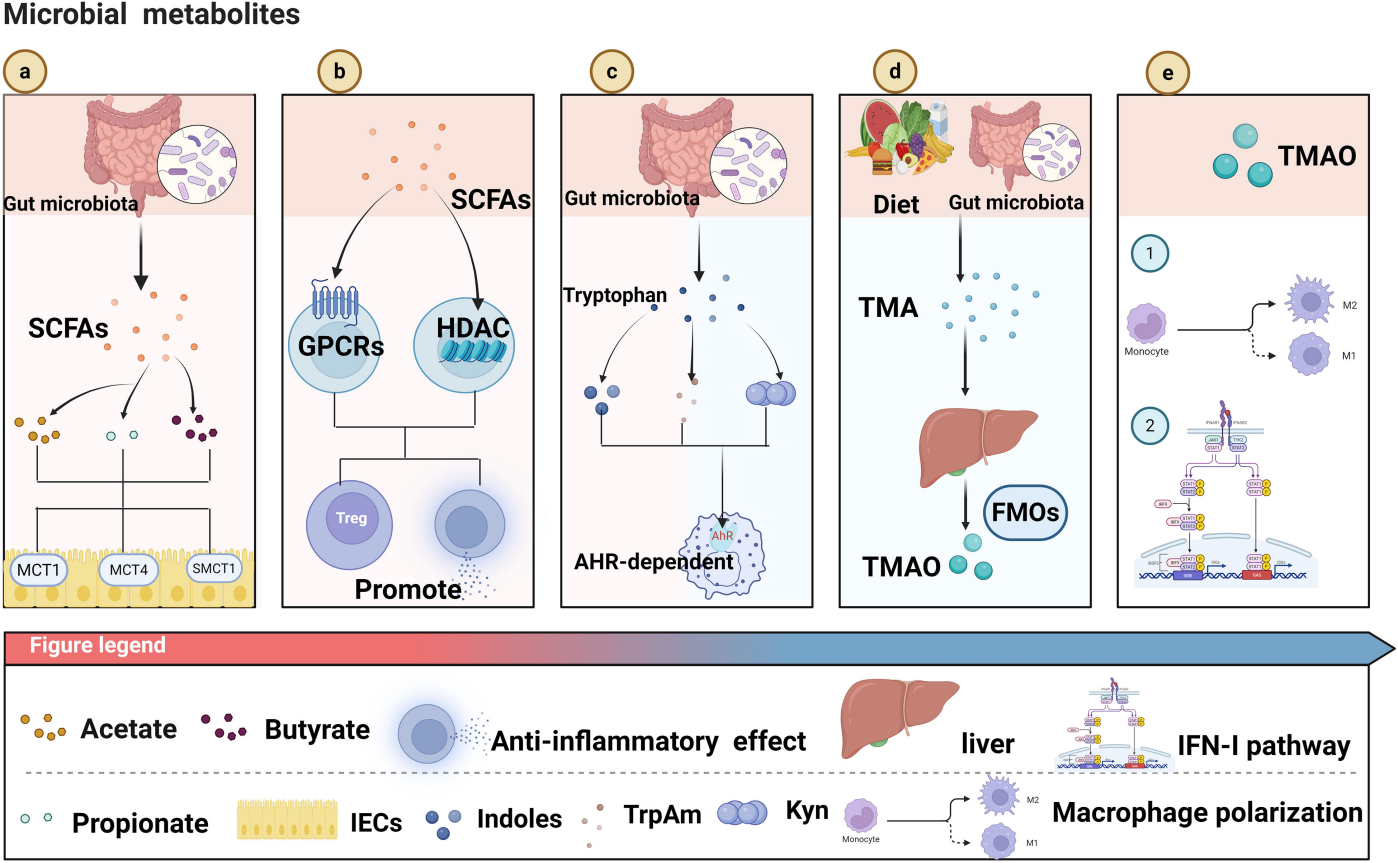
**(a)** Gut microbiota produce short-chain fatty acids (SCFAs, including acetate, propionate, butyrate), which are transported into intestinal epithelial cells (IECs) via transporters like MCT1, MCT4, and SMCT1. **(b)** SCFAs act on G protein-coupled receptors (GPCRs) and inhibit histone deacetylases (HDACs), promoting the generation of regulatory T cells (Treg) and exerting anti-inflammatory effects. **(c)** Gut microbiota metabolize tryptophan into indoles and other metabolites, which activate the aryl hydrocarbon receptor (AhR) - dependent pathway to regulate immune responses. **(d)** Dietary components are metabolized by gut microbiota into trimethylamine (TMA), which is further converted into trimethylamine-N-oxide (TMAO) by flavin-containing monooxygenases (FMOs) in the liver. **(e)** TMAO influences macrophage polarization (into M1 or M2 subtypes) and modulates the interferon-1 (IFN-1) pathway, impacting immune function. Created in https://BioRender.com.

In the 19th century, scientists gradually acknowledged the presence of abundant and diverse microbial communities in the human body. These microbial communities are essential for maintaining health and regulating numerous physiological processes (Consortium, 2012).The gut microbiota, a key regulator of host metabolism, immune function, and barrier integrity, has been closely associated with the development of various cancers. It influences tumor growth and metastasis by affecting inflammatory responses, microbial metabolites, and the immune microenvironment (Zhu et al., 2020). In contrast, intratumoral microbiota, residing within tumor tissues and their microenvironment, have a more direct impact on the biological behavior, metabolic state, and immune landscape of tumor cells, thereby contributing more directly to tumor immune evasion and therapeutic responses (Lam et al., 2021; Zhu et al., 2023c). Furthermore, diverse metabolites impact the tumor microenvironment and the metabolic reprogramming of cancer cells. SCFAs, tryptophan-derived metabolites, and trimethylamine N-oxide (TMAO) are among the most extensively studied microbial metabolites and have been shown to play key roles in cancer development and immunotherapy (**Figure 1**) (**Table 1**).

**Table 1:** Summary of microbial metabolite effects.

Metabolite	Cancer type	Cell type	Target	Model	Reference
Fru-2,6-BP	GC	MKN45,NUGC3	PFKFB3	human gastric cancer cell lines	(Lei et al., 2021)
Butyrate	CRC	HCT116,HT-29	PKM2	CRC cell lines CRC xenograft model	(Li et al., 2018)
Butyrate	CRC	HCT116,HT29,LOVO,HCT8	HDAC3,Akt,ERK1/2	CRC cell lines CRC xenograft model	(Li et al., 2017)
Methionine	CRC	CT26,CD8^+^ T	YTHDF1,PD-L1,VISTA	CRC cell lines CRC xenograft model	(Li et al., 2023)
NaB	CRC	HCT-116,Caco-2	NCOA4,FTH1,Fe²^+^	CRC & normal colon cell lines	(Liu et al., 2025)
BHB	DLBCL	CAR T cells	Metabolic pathways of CAR T cells	Co-culture of A20 cell line with CAR T cells	(Liu et al., 2024)
SCFAs	MM,PAC	CTLs,B16F10,PAN02	mTOR,HDACs	Co-culture of T cells and CAR T cells with SCFAs	(Luu et al., 2021)
Lactate	HCC,MM	B16,Hepa1-6,CD8^+^ T	B7-H3,H3K18la	Co-culture of B16 and Hepa1–6 cells with lactate	(Ma et al., 2025)
TMAO	PAC	CD8^+^ T, Mφ, PCCs	anti-PD-1 antibody	mPCC xenograft	(Mirji et al., 2022)
Cholesterol	LC	A549,95D	AKT	A549,95D	(Mo et al., 2022)
Cystine	NSCLC	A549	Glutaminase	Mouse NSCLC xenograft model	(Muir et al., 2017)
Isobutyric acid	CRC	CT26,CD8^+^ T	PD-1	Mouse colorectal cancer cells–T cells co-culture system	(Murayama et al., 2024)
Glutamine	BC	MDSCs	Glutamine metabolism pathway	Mouse breast cancer xenograft model	(Oh et al., 2020)
Butyrate	CRC	HCT116,HT-29,Caco-2	p21,ERK1/2,c-Myc	HCT116,HT-29,Caco-2	(Oncel et al., 2024)
Methionine	MM	CD4^+^ T ,B16F10	PD-1	Mouse melanoma xenograft model	(Pandit et al., 2023)
Butyrate	CRC	HCT-116,Caco-2	Akt/ERK	Mouse colorectal cancer xenograft model	(Li et al., 2017)
Glutamate	Cancer	Th17 , Th1	GLS	Mouse CD4^+^ T cells *in vitro* differentiation assay	(Johnson et al., 2018)

### 2.1 SCFAs

SCFAs contain one to six carbon atoms, including acetate, propionate, and butyrate. Their concentrations and relative proportions are influenced by multiple factors, including diet composition, microbiota diversity, and the host’s physiological state (Goffredo et al., 2016; Oliver et al., 2024). SCFAs are primarily produced by the gut microbiota via anaerobic fermentation of dietary fibers and other fermentable carbohydrates (Baxter et al., 2019). Additionally, they can be generated through specific metabolic pathways: acetate is produced by most anaerobic bacteria via the acetyl-CoA pathway (Schütze et al., 2020); propionate is mainly synthesized through the succinate or propionyl-CoA pathways (Reichardt et al., 2014); and Clostridium species produce butyrate via condensation reactions involving acetyl-CoA (Duncan et al., 2002). In addition, certain proteins can be converted under specific conditions into branched-chain SCFAs (Wang et al., 2023b). SCFAs are absorbed by colonic epithelial cells via passive diffusion and carrier-mediated transport involving monocarboxylate transporters (MCT1 and MCT4) and the sodium-coupled monocarboxylate transporter (SMCT1) (Kekuda et al., 2013; Nedjadi et al., 2014). As a primary energy source (Donohoe et al., 2011),SCFAs maintain intestinal barrier integrity (Peng et al., 2009) while regulating hepatic metabolism (Zheng et al., 2023), enhancing insulin sensitivity (Gao et al., 2009)and promoting metabolic homeostasis (Tingstad et al., 2025). In addition to serving as energy substrates, SCFAs participate in immune regulation by activating G-protein–coupled receptors (GPR41, GPR43, and GPR109A) (Thangaraju et al., 2009; Kim et al., 2013) and inhibiting HDAC activity (Furusawa et al., 2013). For example, butyrate activates the GPR43 receptor, upregulates the expression of immunoregulatory factors in dendritic cells (DCs), and exhibits marked anti-inflammatory and tissue-repair effects in colitis models (Xiu et al., 2020). Propionate can directly act on γδ T cells, modulating their immune responses by inhibiting HDAC activity and thereby reducing the production of IL-17 and IL-22 (Dupraz et al., 2021).

Butyrate, as the most extensively studied metabolite among SCFAs, exerts multifaceted effects on tumor progression, immune regulation, and therapeutic responses (Che et al., 2023).In terms of tumor progression, the role of butyrate has been most extensively investigated in Colorectal Cancer(CRC) (Okumura et al., 2021). Li et al. reported that exposing colorectal cancer cell lines to varying concentrations of butyrate for 24 hours significantly reduced their migratory capacity. Mechanistically, butyrate inhibited colorectal cancer cell motility by suppressing the Akt/ERK signaling pathway in an HDAC3-dependent manner (Li et al., 2017). In contrast, Wu et al. found that gut butyrate levels were decreased in mice colonized with Fusobacterium nucleatum(*F. nucleatum*). Mechanistically, sodium butyrate (NaB) activated the AMPK signaling pathway, inducing cell cycle arrest and mitochondrial morphological damage in HCT116 and DLD-1 cells, which impaired ATP and Reactive Oxygen Species(ROS) production and consequently inhibited cancer cell proliferation (Wu et al., 2024). Moreover, the effects of butyrate are concentration-dependent. Treatment of human macrophages with 0.1 mM sodium butyrate inhibited LPS-induced production of the pro-inflammatory cytokine TNF-α, exhibiting anti-inflammatory effects, whereas 10 mM sodium butyrate promoted macrophage cell death and enhanced IL-1β production, displaying pro-inflammatory activity (Jiang et al., 2025). In addition, the sensitivity to butyrate varies among different types of colorectal cancer cells (Oncel et al., 2024). Recent studies have also revealed a link between butyrate and ferroptosis. NaB promotes ferritinophagy in HCT-116 and Caco-2 cells, downregulates FTH1, upregulates NCOA4, and increases intracellular Fe²^+^ levels, thereby inducing ferroptosis and exhibiting potential anti-colorectal cancer effects (Liu et al., 2025). In terms of immune regulation, butyrate also exerts multiple effects. Firstly, it plays a role in maintaining the balance between Regulatory T cell (Treg) and Th17 cells. In a rat model of colitis, administration of sodium butyrate increased the levels of peripheral blood Treg cells and plasma concentrations of anti-Th17 cytokines (IL-10 and IL-12), while suppressing IL-17 levels in both plasma and colonic mucosa, thereby protecting the colonic mucosa from inflammatory damage (Zhang et al., 2016). In the context of anti-tumor immunotherapy, studies have shown that butyrate can enhance tumor metabolism by activating carnitine palmitoyltransferase 1A (CPT1A)-mediated Fatty Acid Oxidation(FAO),thereby suppressing CD8^+^ T cell activity and promoting resistance of CRC to anti-programmed cell death protein 1 (anti-PD-1). In CPT1A knockdown models, combined treatment with anti-PD-1 and butyrate restored CD8^+^ T cell infiltration and enhanced anti-tumor efficacy (Zhu et al., 2025a). In contrast, Luu et al. demonstrated *in vitro* that treatment of cytotoxic T lymphocytes (CTLs) and chimeric antigen receptor (CAR) T cells with butyrate enhanced the function of mTOR, a central cellular metabolic sensor, while inhibiting class I HDAC activity. This reprogramming significantly increased the production of effector molecules such as CD25, IFN-γ, and TNF-α, and markedly improved the anti-tumor activity of antigen-specific CTLs and ROR1-targeting CAR T cells in syngeneic murine models of melanoma and pancreatic cancer (Luu et al., 2021). Taken together, SCFAs not only have potential as predictive biomarkers for immune responses, but their effects may also be bidirectional, depending on the type of immune checkpoint inhibitor (ICIs), the host microbiota, and the cancer cell type and immune context.

### 2.2 Tryptophan metabolites

Tryptophan is an essential amino acid that serves not only as a building block for protein synthesis, supporting cell proliferation, growth, and tissue repair, but also as a precursor for diverse metabolic pathways, generating bioactive molecules and contributing to the maintenance of redox balance by the gut microbiota. The gut microbiota metabolizes tryptophan into diverse bioactive compounds, including indole derivatives, tryptamine, and Kynurenine (Kyn). These metabolites regulate host immunity, preserve intestinal barrier integrity, and influence tumors via aryl hydrocarbon receptor (AhR)-dependent and AhR-independent pathways (Hou et al., 2023). The AhR is a ligand-activated transcription factor, and bacterially derived tryptophan metabolites, including indole-3-acetic acid (IAA) and indole-3-propionic acid (IPA), function as its agonists. Wen et al. demonstrated in a mouse model that gut dysbiosis significantly decreased IAA and IPA levels, suppressing AhR signaling, upregulating Sterol regulatory element-binding protein 2 (SREBP2) expression, and promoting hepatocarcinogenesis.

Supplementation with *Lactobacillus reuteri*, a bacterium that produces AhR agonists, downregulated SREBP2 and markedly inhibited liver cancer development. These findings indicate that the gut microbiota modulates the AhR–SREBP2 signaling axis via tryptophan metabolites, thereby promoting the initiation of hepatocarcinogenesis (Chen et al., 2022b). Moreover, the tryptophan metabolite indole-3-lactic acid promotes DCs to secrete IL12 by enhancing Histone H3 lysine 27 acetylation (H3K27ac) modification at the IL12 enhancer, thereby activating the antitumor functions of CD8^+^ T cells. In addition, it modulates chromatin accessibility to suppress the cholesterol metabolism–related gene *Saa3* expression in CD8^+^ T cells, thereby enhancing the activity of tumor-infiltrating CD8^+^ T cells (Zhang et al., 2023). These findings underscore the pivotal role of tryptophan and its metabolites in modulating antitumor immune responses.

### 2.3 TMAO

TMAO is a bioactive metabolite that can be acquired directly from the diet or generated by the gut microbiota via the conversion of dietary choline, carnitine, and phospholipids into trimethylamine (TMA), which is subsequently oxidized in the liver by flavin-containing monooxygenases (FMOs) to form TMAO (Romano et al., 2015). In recent years, TMAO has been implicated in developing and progressing multiple diseases, including cardiovascular disorders, metabolic syndrome, and certain cancers, where it modulates immune cell function and metabolic pathways to influence tumor growth and metastasis (Senthong et al., 2024). For instance, Gauri et al. employed non-targeted liquid chromatography–tandem mass spectrometry (LC–MS/MS)–based metabolomics and demonstrated that TMAO enhances antitumor immunity against pancreatic ductal adenocarcinoma (PDAC). The underlying mechanisms mainly involve promoting the polarization of tumor-associated macrophages (TAMs) toward an immunostimulatory phenotype, thereby enhancing their antitumor activity, and activating effector CD8^+^ T cells within the tumor microenvironment, which increases their cytotoxicity and antitumor functions. TMAO also activates the type I interferon signaling pathway, essential for modulating antitumor immunity, and exerts its immunostimulatory effects in a pathway-specific manner. Furthermore, in PDAC mouse models, the combination of TMAO and anti–PD-1 therapy markedly reduced tumor burden and extended survival, demonstrating a strong synergistic antitumor effect (Mirji et al., 2022).

## 3 Tumor metabolic reprogramming and its regulation by microbiota

### 3.1 Reprogramming of glucose metabolism

Glucose, the primary energy source for cells, generates ATP through glycolysis, the tricarboxylic acid (TCA) cycle, and oxidative phosphorylation (OXPHOS) to supply energy for bodily functions. In healthy cells with enough oxygen, glucose is broken down via glycolysis to produce pyruvate, which then enters the mitochondria to go through the TCA cycle and OXPHOS, efficiently generating ATP. This pathway is the main and most effective way for cells to produce energy (Grubelnik et al., 2025). When oxygen is scarce, cells switch to anaerobic glycolysis, converting pyruvate into lactate to keep ATP production. Although less efficient, this shift allows cells to adapt to oxygen shortages quickly. Additionally, once glucose enters the cytoplasm, it can be directed into several metabolic pathways, such as the hexosamine biosynthetic pathway (HBP), the pentose phosphate pathway (PPP), and serine biosynthesis. These pathways produce key intermediates or precursors—nucleotides, amino acids, and methyl groups—essential for biosynthesis and cellular signaling regulation. Therefore, maintaining a balance in glucose metabolism is vital for cell survival (Gkiouli et al., 2019). Tumor cell glucose metabolism greatly differs from that of normal cells. In the early 20th century, Warburg proposed the significant hypothesis that cancer cells tend to convert glucose into lactate even when oxygen is plentiful, a phenomenon later called the “Warburg effect” (Mathew et al., 2024). Initially, this metabolic shift was thought to result from impaired mitochondrial function in cancer cells. However, later studies challenged this view. Using radioisotope tracing experiments, Wein and colleagues showed that tumor cells can still oxidize glucose to carbon dioxide, indicating their OXPHOS capacity remains similar to normal cells (Weinhouse and Wenner, 1956). Furthermore, in the hypoxic core of tumors, cancer cells reduce OXPHOS activity while increasing glycolysis to sustain energy production. These findings suggest that mitochondrial problems do not cause the Warburg effect but instead reflect active metabolic reprogramming by cancer cells. This shift supports cancer cell survival and adaptation and promotes metastasis by aiding epithelial–mesenchymal transition (EMT), increasing angiogenesis, and enabling tumor spread to distant organs.

Although different cancer types show varied patterns of glucose reprogramming, they generally follow similar strategies to control glucose intake and key glycolytic steps to meet the high energy demands of rapid growth. These common mechanisms primarily include the regulation of enzyme expression, modulation by hypoxia-inducible factor 1 (HIF-1), control of transporter activity, and activation of oncogenes and associated signaling pathways and Metabolic adaptation. In terms of enzyme expression, Fructose-2,6-bisphosphatase 3 (PFKFB3) acts as an activator, indirectly influencing phosphofructokinase-1 (PFK-1), affecting glycolytic rate. In cancer cells, increased PFKFB3 levels activate the NF-κB pathway, promoting EMT and enhancing the migratory capacity of gastric cancer (GC) cells (Lei et al., 2021). In low oxygen conditions, HIF-1 coordinates metabolic changes along with other mechanisms. Normally, HIF-1 is hydroxylated and quickly degraded when oxygen is plentiful. Under hypoxia, its degradation is prevented, allowing HIF-1 to accumulate in cancer cells (Marxsen et al., 2004). HIF-1 increases lactate dehydrogenase A (LDHA) and Monocarboxylate transporter 4 (MCT4) levels, promoting the conversion of pyruvate to lactate and its export (Yamaguchi et al., 2023). It also upregulates Glucose transporter 1 (GLUT1) and hexokinase 2 (HK2), enhancing glucose uptake and its conversion to glucose-6-phosphate, thereby increasing lactate production in colorectal cancer cells (Shen et al., 2020). In terms of transporters, monocarboxylate transporter 1 (MCT1) and MCT4 are primarily responsible for the transmembrane transport of lactate in and out of tumor cells, leading to an acidic tumor microenvironment that favors cancer cell metastasis and immune evasion. Experimental evidence provides strong support for this mechanism: inhibition of MCT4 or the use of MCT inhibitors reduces the ability of PDAC cells to uptake lactate, while knockout of MCT4 markedly suppresses the migratory potential of PDAC cells (Kong et al., 2016). Moreover, Aberrant activation of oncogenes and key signaling pathways also plays a pivotal role in the reprogramming of glucose metabolism. Oncogenic mutations in *KRAS* and *BRAF* upregulate the expression of GLUT1 and other GLUT isoforms, thereby promoting their translocation to the plasma membrane and enhancing glucose uptake capacity (Beg et al., 2017). Moreover, hyperactivation of Yes-associated protein (YAP) in cooperation with the loss of *p53* function markedly increases the enrichment of glucose transporter 3 (GLUT3) on the plasma membrane, further facilitating transmembrane glucose transport and augmenting the energy supply and metabolic activity of cancer cells (Wang et al., 2015). Collectively, by modulating both the expression and subcellular localization of glucose transporters, these oncogenic alterations synergistically drive the reprogramming of glucose metabolism in malignant cells. Multiple signaling pathways are concurrently involved in the reprogramming of glucose metabolism (Ciscato et al., 2021). The PI3K/AKT and mTORC1 signaling pathways can upregulate the expression of key rate-limiting enzymes, glucose-6-phosphate dehydrogenase (G6PD) and ribose-5-phosphate isomerase A (RPIA), by inducing the activation of SREBP1, thereby fulfilling the metabolic demands of cancer cells for nucleotides and redox homeostasis (Simcox and Lamming, 2022). Certain gain-of-function mutations in *p53* promote the plasma membrane localization of GLUT1 by modulating the RhoA/ROCK signaling pathway, thereby enhancing transmembrane glucose transport (Zhang et al., 2013). Collectively, these signaling pathways increase glucose uptake and utilization in cancer cells, supporting high metabolic activity and sustained proliferative capacity. Finally Metabolic adaptation refers to the ability of cancer cells to adjust their metabolic pathways under conditions of glucose or nutrient limitation to maintain essential supply of metabolites and energy. For instance, cancer cells can activate gluconeogenic pathways to generate intermediates of glycolysis that support metabolic activities. The mitochondrial isoform of phosphoenolpyruvate carboxykinase (PEPCK-M) is upregulated in breast and lung cancer cells, and both its expression and activity are further enhanced under glucose-restricted conditions, contributing to the maintenance of metabolic homeostasis and enabling cancer cells to adapt to nutrient stress (Hsu et al., 2023).These mechanisms work together to reprogram glucose metabolism in cancer, meeting the energy needs of fast-growing, invasive, and metastatic cells. 

**Figure 2:**
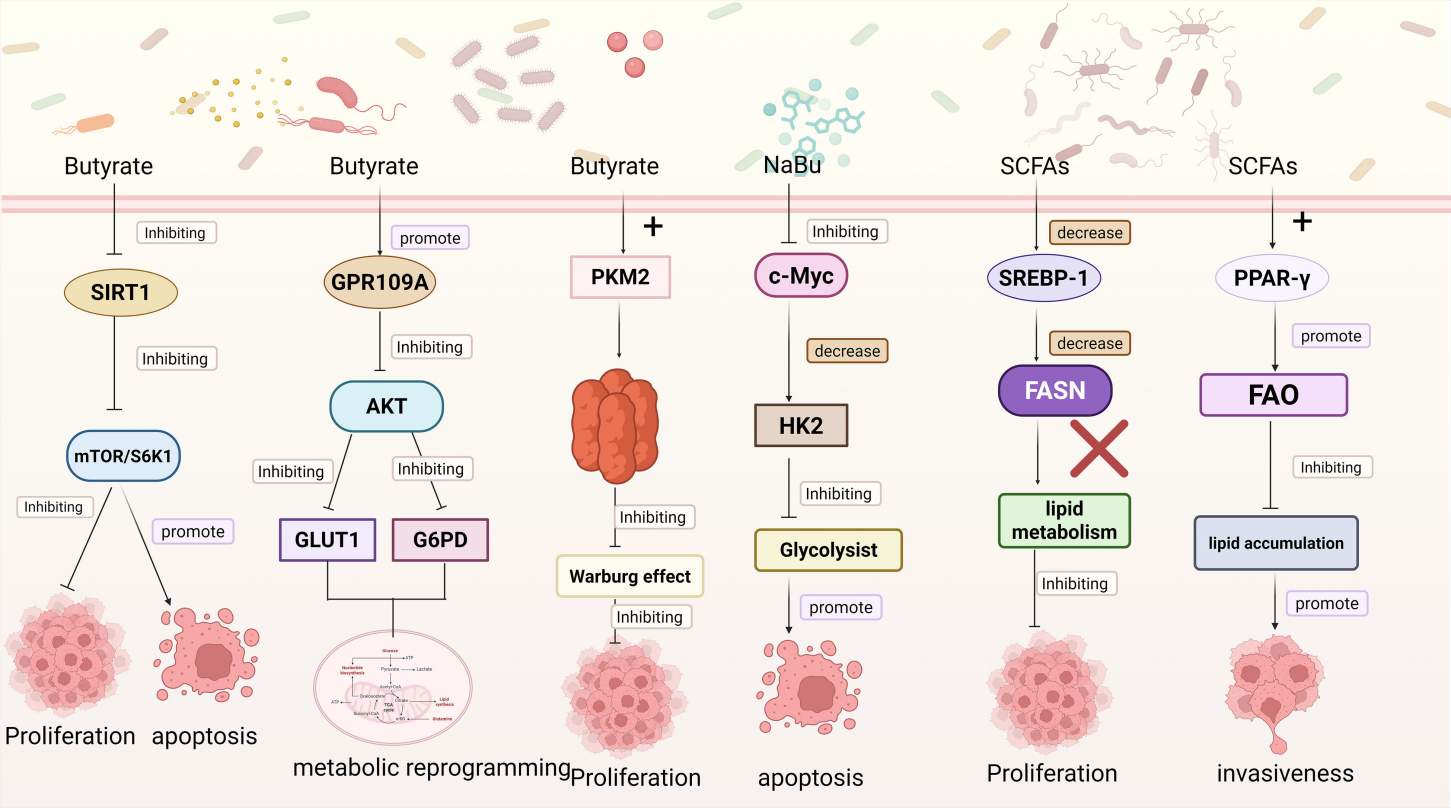
This figure depicts multiple pathways through which microbial metabolites modulate cancer cell functions: Butyrate inhibits SIRT1, suppressing the mTOR/S6K1 pathway. This inhibition restrains cancer cell proliferation and promotes apoptosis; Butyrate activates GPR109A, which inhibits the AKT pathway. This further downregulates GLUT1 and G6PD, inducing metabolic reprogramming in cancer cells. Butyrate interacts with PKM2 to inhibit the Warburg effect, thus suppressing cancer cell proliferation. Sodium butyrate (NaBu) inhibits c-Myc, leading to a decrease in HK2. This inhibits glycolysis and promotes cancer cell apoptosis; SCFAs downregulate SREBP-1 1, reducing FASN expression and inhibiting lipid metabolism to suppress cancer cell proliferation; SCFAs activate PPAR-γ, promoting fatty acid oxidation (FAO). This inhibits lipid accumulation and enhances cancer cell invasiveness. Created in https://BioRender.com.

Beyond the intrinsic metabolic reprogramming mechanisms of cancer cells, extrinsic factors can also markedly influence glucose metabolism, among which microbial metabolites have attracted considerable attention due to their broad biological activities. Like the intrinsic metabolic reprogramming of cancer cells, microbial metabolites exhibit certain shared regulatory features, which can be broadly categorized into two mechanisms: one mediated through signaling pathways to modulate metabolism, and the other directly targeting key metabolic enzymes to redirect metabolic flux. Signal-Mediated Control of Glucose Metabolism: Among various SCFAs, butyrate has attracted particular attention due to its pronounced antitumor activity, with part of its mechanism exerted through modulation of key signaling pathways in cancer cells, thereby impacting their metabolic processes. For instance, the mTOR/S6K1 pathway serves as a pivotal regulatory axis of cellular metabolism and growth. Inactivation of this pathway suppresses protein synthesis and energy metabolism, thereby impairing the proliferative capacity of cancer cells and promoting apoptosis. SIRT1, a NAD^+^-dependent deacetylase, is involved in glucose metabolism, fatty acid oxidation, and mitochondrial function in cancer cells. By activating or repressing key transcription factors such as PGC-1α and FOXO, Sirtuin 1 (SIRT1) orchestrates metabolic reprogramming in cancer cells to adapt to fluctuations in energy availability (Zhang et al., 2025). Moreover, butyrate has been shown to downregulate SIRT1 expression and suppress the activity of the mTOR/S6K1 signaling pathway in colorectal cancer HCT116 cells, thereby modulating tumor metabolism, inhibiting cell growth, and promoting apoptosis (Cao et al., 2019). Meanwhile, butyrate activates GPR109A on the surface of CRC, thereby inhibiting the AKT signaling pathway and subsequently downregulating the expression of glucose transporter GLUT1 and the key metabolic enzyme G6PD. This mechanism reduces glucose uptake, glycolytic flux, and lactate production, mediating metabolic reprogramming of cancer cells, restricting energy supply, and ultimately suppressing proliferation (Geng et al., 2021).In addition to regulating glucose metabolism through signaling pathways, butyrate can also directly modulate the activity of key glycolytic enzymes. Pyruvate kinase M2 (PKM2), a critical rate-limiting enzyme in the final step of glycolysis, catalyzes the conversion of phosphoenolpyruvate (PEP) to pyruvate with concomitant generation of ATP (Jin et al., 2025). Quantitative proteomics has revealed that butyrate directly targets PKM2 to reprogram the metabolism of colorectal cancer cells. Upon binding to PKM2, butyrate promotes the formation of a highly active tetrameric conformation, thereby enhancing its catalytic efficiency and suppressing the Warburg effect in colorectal cancer cells. This results in a reduction of glycolytic intermediates and accumulation of pyruvate, while limiting the availability of precursors for nucleotide biosynthesis, ultimately inhibiting colorectal cancer cell proliferation (Li et al., 2018). On the other hand, butyrate can also indirectly influence the expression of glycolysis-related enzymes by regulating upstream transcription factors. As a critical metabolic regulator, c-Myc broadly governs the transcription of genes involved in glycolysis, lipid metabolism, and other metabolic pathways. Studies have shown that NaBu suppresses lactate release and glucose uptake in Hepatocellular carcinoma (HCC) cells *in vitro* and reduces lactate production *in vivo* in mice. This effect is mediated through inhibition of the c-Myc signaling pathway, leading to downregulation of HK2, thereby suppressing glycolysis, restricting energy supply, and inducing apoptosis in cancer cells (Yu et al., 2022) (**Figure 2**).

In summary, this section delineates the mechanistic regulation of glucose metabolism in cancer cells, encompassing both intrinsic metabolic reprogramming and modulation by microbial metabolites. Cancer cells sustain high energy demands and biosynthetic activity by regulating the expression of key glycolytic enzymes, controlling HIF-1, modulating transporter expression, activating oncogenes and associated signaling pathways, and implementing metabolic adaptations. Concurrently, microbial metabolites influence glucose metabolism by modulating signaling pathways and the activity of rate-limiting enzymes. Overall, these mechanisms work in concert to reprogram glucose flux.

### 3.2 Lipid metabolism and amino acid metabolism

Lipids are essential components of biological membranes, help regulate signal transduction, and play crucial roles in energy storage (Dietrich et al., 2001). Lipid metabolism mainly involves two central processes: fatty acid synthesis and fatty acid oxidation (FAO). It intersects with various metabolic pathways, including the pentose phosphate pathway, the malate–oxaloacetate shuttle, cholesterol biosynthesis, and triglyceride synthesis. These interconnected metabolic networks collectively ensure that cells fulfil their essential demands for lipid biosynthesis and energy provision (Pascual et al., 2024).Cancer cells establish a multilayered and interactive lipid metabolic network through the regulation of enzyme expression, the signaling functions of ROS, the mediation of lipid transporters, and the modulation of intracellular signaling pathways. For example, At the enzymatic level, fatty acid synthase (FASN) catalyzes the condensation of acetyl-CoA and malonyl-CoA to generate long-chain fatty acids. FASN is markedly upregulated in cancer-associated fibroblasts (CAFs), and its knockdown decreases fatty acid synthesis along with Vimentin and E-cadherin expression in CAFs, consequently attenuating the invasive and migratory potential of DLD1 cells (Gong et al., 2020). Similarly, Carnitine palmitoyl transferase 1 (CPT1) catalyzes the conversion of long-chain fatty acyl-CoAs to acylcarnitine, facilitating their transport across the mitochondrial inner membrane for subsequent β-oxidation. Studies have demonstrated that CPT1A is markedly upregulated in CRC cells under suspension culture and in metastatic lesions in mouse models. CPT1A knockdown reduces pulmonary metastasis in CRC-bearing mice, induces phenotypic alterations in cancer cells, and diminishes their metastatic potential (Wang et al., 2018). Moreover, FAO generates ROS and their accumulation in CRC cells can activate the MAPK signaling pathway, thereby promoting EMT and enhancing cancer cell invasion and migration (Ishiguro et al., 2017; Wang et al., 2019). In terms of transporters, In peritoneal metastases of gastric cancer, apolipoprotein C2 (APOC2) is markedly upregulated and promotes EMT in cancer cells via the CD36-mediated PI3K/Akt/mTOR signaling pathway, thereby enhancing their migratory capacity (Wang et al., 2021). Finally, signaling pathways also play a crucial role in lipid metabolism. Reducing intracellular free cholesterol can activate the PI3K/Akt signaling pathway, induce mitochondrial dysfunction, and enhance lung cancer cells’ invasiveness and metastatic potential (Mo et al., 2022). PKM2 physically interacts with sterol regulatory element-binding protein 1c (SREBP-1c) through biochemical association. Downregulation of PKM2 decreases the expression of SREBP-1c by inactivating the AKT/mTOR signaling pathway, thereby directly suppressing the transcription of the key lipogenic gene FASN and modulating lipid metabolism. This suppression of lipid synthesis ultimately reduces tumor growth, providing a novel therapeutic target and mechanistic basis for the treatment of bladder cancer (Tao et al., 2019).

Lipid metabolism is not only a fundamental process that fulfills the energy demands of tumor cells but also serves as a pivotal hub for immune regulation. Nasopharyngeal carcinoma cells promote the differentiation and immunosuppressive function of Tregs through the CD70–CD27 interaction, thereby attenuating anti-tumor immune responses. Blocking CD70 signaling can restore the cytotoxic activity of CD8^+^ T cells. In animal models, the combination of a CD70 antibody with anti–PD-1 therapy exhibits a pronounced synergistic anti-tumor effect, markedly enhancing immunotherapeutic responses. Mechanistic studies further reveal that the CD70–CD27 axis remodels lipid metabolism–related signaling networks in both Tregs and naïve CD4^+^ T cells, involving mitochondrial function, cholesterol homeostasis, and fatty acid metabolic reprogramming, highlighting a critical metabolic crosstalk between lipid metabolism and immune regulation (Gong et al., 2023).Similarly, This study demonstrates that CD36-mediated uptake of OxLDL in CD36^+^ CAFs induces macrophage MIF expression through the lipid peroxidation/p38/CEBP transcriptional axis. Secreted MIF, in turn, recruits CD33^+^ myeloid-derived suppressor cells (MDSCs) via the CD74 receptor, thereby enhancing their immunosuppressive activity. Notably, combined treatment with a CD36 inhibitor and anti-PD-1 immunotherapy effectively restored anti-tumor T cell responses, exhibiting a synergistic therapeutic effect. These findings underscore the pivotal role of specific CAF subsets in modulating the crosstalk between the tumor microenvironment and the immune system (Zhu et al., 2023a).Overall, lipid metabolism regulates the functional activity of both T cells and CAFs, thereby shaping the responsiveness to immune checkpoint blockade (ICB) therapy. Notably, machine learning–based research strategies that construct predictive models using lipid metabolic features provide an innovative and promising approach for evaluating immunotherapeutic responses. Chen et al. conducted a systematic analysis of lipid metabolic characteristics based on data from The Cancer Genome Atlas (TCGA) lung adenocarcinoma (LUAD) cohort and found that the aberrant expression of lipid metabolism–related genes was closely associated with immune cell infiltration patterns and responses to immunotherapy. On this basis, the research team established a Lipid Metabolism Score (LMS) system and identified MK1775 as a potential sensitizing agent that targets lipid metabolism to enhance the efficacy of anti–PD-1 therapy. Mechanistically, MK1775 was shown to suppress the PI3K/AKT/mTOR signaling pathway, thereby downregulating FASN–mediated fatty acid synthesis to inhibit fatty acid oxidation in TAMs. Concurrently, MK1775 activated the interferon regulatory factor (IRF) pathway, promoting the secretion of CXCL10 and CXCL11, which facilitated CD8^+^ T-cell infiltration into the tumor microenvironment. Both *in vitro* and *in vivo* experiments demonstrated that the combination of MK1775 and anti–PD-1 antibody markedly suppressed tumor growth by enhancing the immune sensitivity of the tumor microenvironment through coordinated metabolic and immunological regulation (Chen et al., 2024; Ping et al., 2025). Interestingly, the amphiphilic nature of lipids, possessing both polar and lipophilic properties, makes them excellent candidates for drug delivery vehicles. A lymph node-targeting lipid nanoparticle (LNP) named 113-O12B efficiently delivers mRNA vaccines and induces robust antigen-specific CD8^+^ T cell responses. In murine tumor models, 113-O12B LNPs carrying OVA or TRP-2 mRNA exhibit significant tumor inhibition, with enhanced efficacy when combined with anti–PD-1 therapy, and elicit durable immune memory (Chen et al., 2022a).

Beyond lipid metabolism, the reprogramming of amino acid metabolism also plays a pivotal role in sustaining tumor growth and regulating immune responses. Amino acids serve as the fundamental building blocks for protein synthesis and play crucial regulatory roles in maintaining cellular metabolic homeostasis, antioxidant defense, and signal transduction (Reid et al., 2018).Amino acid metabolism primarily encompasses three major aspects: first, amino acids act as nitrogen donors for the biosynthesis of nucleotides, neurotransmitters, and other non-protein nitrogenous compounds (Zhu et al., 2017);second, they provide carbon skeletons and energy to fuel the TCA cycle (Muir et al., 2017);and third, they maintain intracellular redox balance and modulate signal transduction (Wolfson et al., 2016).In tumor cells, amino acid metabolism is systematically reprogrammed to meet the elevated demands for energy and biosynthetic precursors associated with high proliferative activity. This reprogramming is characterized not only by aberrant expression of key metabolic enzymes but also by the remodeling of amino acid transporters and signaling pathways, ultimately influencing tumor metabolic homeostasis and the immune microenvironment. For example, SHMT2, a key enzyme in the serine–glycine–one-carbon (SGOC) metabolic pathway, generates methyl donors, participates in nucleotide biosynthesis, and promotes NADPH production. Studies have demonstrated that SHMT2 is markedly upregulated in CRC, thereby promoting tumor growth; conversely, SHMT2 knockdown induces G0/G1 phase arrest and reduces the S-phase fraction in CRC cells, accompanied by downregulation of CCND1 and CDK2 expression and upregulation of the inhibitory protein *p27* (Cui et al., 2022). Similarly, the glutamine transporter ASCT2 is highly expressed in various cancers, and its aberrant activation in neuroblastoma is closely associated with poor prognosis. Activating transcription factor 4 (ATF4) and N-Myc cooperatively bind to the ASCT2 promoter, enhancing its transcriptional activity and thereby increasing glutamine uptake to supply energy and carbon sources for tumor cells. Inhibition of ASCT2 effectively blocks glutamine utilization and suppresses tumor cell proliferation and migration. Likewise, branched-chain amino acid transaminase 1 (BCAT1) promotes cell proliferation and maintains redox homeostasis by activating the PI3K/AKT/mTOR signaling pathway (Shu et al., 2021). Collectively, these findings suggest that tumor cells achieve dual regulation of energy supply and signaling networks through modulation of amino acid metabolism–related enzymes and transporters. Beyond promoting tumor metabolic adaptation, amino acid metabolic reprogramming also profoundly shapes the immune microenvironment. The tryptophan–kynurenine–aryl hydrocarbon receptor (Trp–Kyn–AhR) axis represents a prototypical immunometabolic suppressive pathway. Indoleamine 2,3-dioxygenase 1 (IDO1) catalyzes the conversion of tryptophan to Kyn, leading to local tryptophan depletion and suppression of mTORC1 activity, while activating the GCN2–eIF2α pathway, thereby impairing the metabolic and effector functions of CD8^+^ T cells. The accumulated Kyn further activates AhR, triggering an immunosuppressive transcriptional program that promotes Treg expansion, enhances IL-10 secretion, and suppresses interferon-β (IFN-β) signaling in DCs. Simultaneously, it induces functional exhaustion of NK and CD8^+^ T cells, collectively dampening antitumor immune responses (Labadie et al., 2019). Anastasaki et al. analyzed single-cell transcriptomic data and employed brain tumor models to reveal that the glutamate signaling pathway is significantly enriched in tumor cells, and glutamate promotes the proliferation of pilocytic astrocytoma (PA) cells. Further mechanistic investigations demonstrated that aberrantly expressed glutamate receptor subunits GRID2 and GRIK3 selectively activate platelet-derived growth factor receptor α (PDGFRα) through Src-mediated signaling, thereby enhancing the activity of the Ras/ERK pathway. Accordingly, suppression of GRID2/GRIK3 or PDGFRA expression markedly attenuates PDGFRα/Ras/ERK pathway activation, inhibits PA cell proliferation, and reduces the growth of xenograft tumors (Anastasaki et al., 2025).

Moreover, amino acid metabolism contributes to the formation of an immunosuppressive microenvironment through intercellular metabolic competition. In breast cancer, tumor cells release large amounts of arginine, providing metabolic substrates for TAMs. These TAMs convert arginine into polyamines, which activate the p53 signaling pathway and, through thymine DNA glycosylase (TDG)-mediated DNA demethylation, regulate the expression of PPAR-γ and pro-tumorigenic genes such as PD-L1 and IL-10. The polarized TAMs subsequently secrete immunosuppressive cytokines and express inhibitory molecules that impair the cytotoxic activity of CD8^+^ T cells. Animal studies have demonstrated that disrupting the arginine–polyamine–TDG axis between tumor cells and TAMs markedly suppresses breast cancer growth (Zhu et al., 2025b).Methionine metabolic imbalance also plays a crucial role in tumor–immune interactions and exerts profound effects on the responsiveness to immunotherapy. In the tumor microenvironment, excessive methionine uptake by cancer cells reduces intracellular methionine levels in CD4^+^ T cells. Methionine deprivation decreases histone H3K79 demethylation (H3K79me2), which in turn downregulates AMPK expression, upregulates PD-1, and impairs the antitumor function of CD4^+^ T cells. AMPK-deficient CD4^+^ T cells exhibit endoplasmic reticulum stress and elevated Xbp1s transcript levels. SLC43A2, the primary methionine transporter on cancer cell membranes, mediates this metabolic competition; its deletion restores methionine metabolism in CD4^+^ T cells. Supplementation with methionine rescues H3K79 methylation and AMPK expression, thereby reducing PD-1 levels. These findings indicate that AMPK serves as a methionine-dependent epigenetic regulator of PD-1 expression in CD4^+^ T cells and functions as a metabolic checkpoint governing T cell exhaustion (Pandit et al., 2023).Similar to this study, feeding mice a methionine-restricted diet markedly enhances antitumor immune responses. Mechanistically, S-adenosylmethionine (SAM), the key methyl donor derived from methionine metabolism, promotes N^6^-methyladenosine (m^6^A) modification of PD-L1 mRNA, thereby increasing its translational efficiency and facilitating tumor immune evasion. The m^6^A reader protein YTHDF1 plays a critical role in this process by promoting the translation of immune checkpoint proteins to sustain an immunosuppressive tumor microenvironment. Methionine restriction or YTHDF1 deletion reduces immune checkpoint expression, restores CD8^+^ T cell function, and exhibits a strong synergistic effect with PD-1 blockade therapy, suggesting that targeting methionine metabolism may represent a promising strategy to enhance the efficacy of immunotherapy (Li et al., 2023).

The above content elucidates the key mechanistic roles of lipid and amino acid metabolism in tumor metabolic reprogramming and immune regulation. Both metabolic processes contribute to sustaining the high proliferative and biosynthetic demands of cancer cells by modulating the expression of key metabolic enzymes, engaging ROS mediated signaling, regulating transporter activity, and integrating multiple intracellular signaling pathways. Concurrently, these metabolic pathways profoundly influence antitumor immune responses through mechanisms involving metabolic competition, signaling pathway modulation, and epigenetic regulation. They govern immune checkpoint expression, T cell function, the activity of CAFs, the recruitment of myeloid-MDSCs, and the polarization of TAMs, collectively reshaping the immunosuppressive tumor microenvironment and determining the efficacy of immunotherapy. Therefore, targeting lipid and amino acid metabolic pathways represents a promising therapeutic strategy and a potential key avenue for enhancing the effectiveness of immunotherapy.

**Figure 3:**
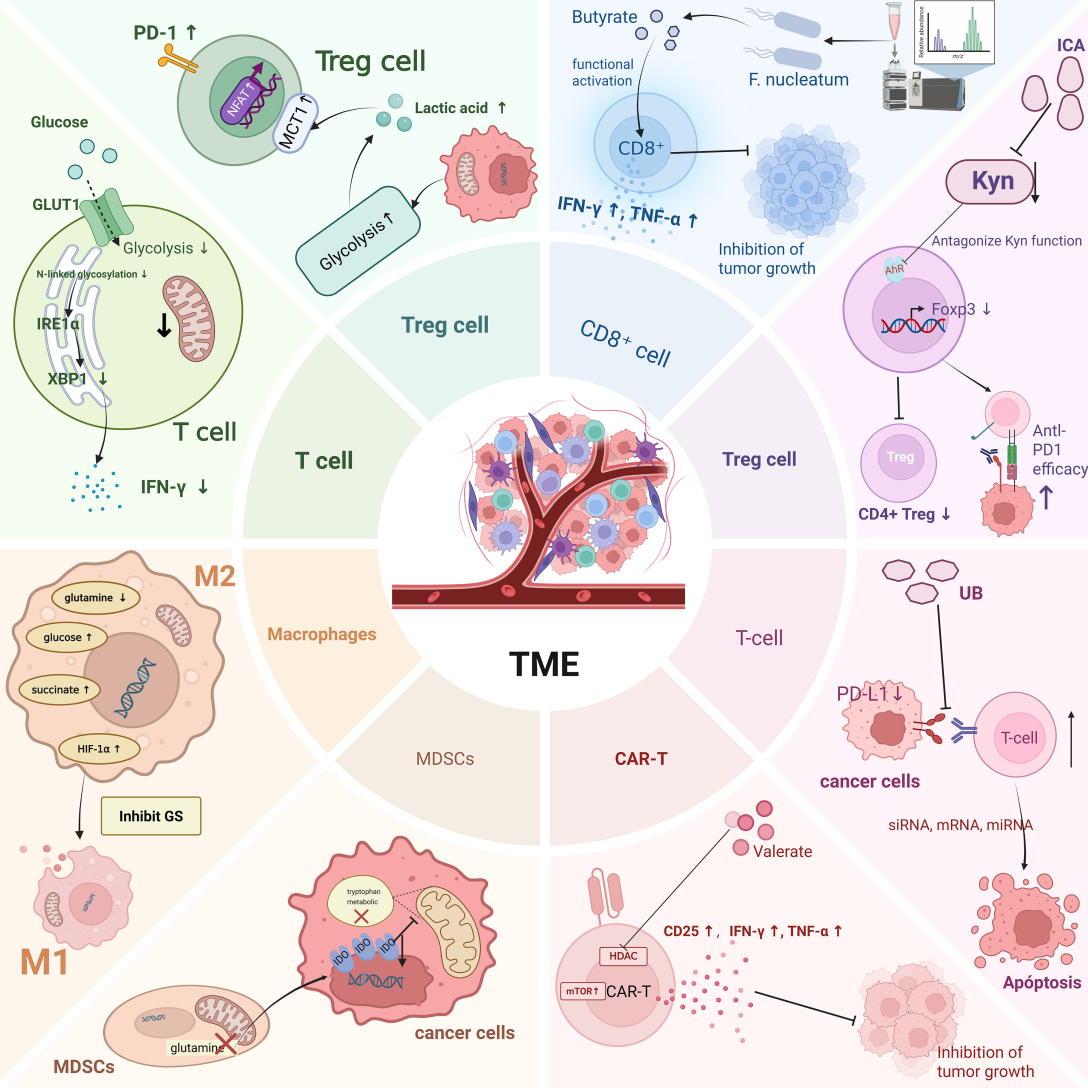
This figure shows metabolic reprogramming within tumor-associated immune cells on the left and the impact of microbial metabolites on the metabolic reprogramming of immune cells on the right. Created in https://BioRender.com.

The mechanisms by which metabolites regulate lipid and amino acid metabolic reprogramming are analogous to those observed in glucose metabolism, primarily exerting their effects through two main routes: directly acting on metabolic enzymes and modulating metabolic pathways via signaling cascades.CPT1A is the rate-limiting enzyme in the FAO process, catalyzing the esterification of long-chain acyl-CoA with carnitine to generate acylcarnitine, thereby enabling fatty acids to traverse the mitochondrial membrane and enter the mitochondrial matrix for β-oxidation. Upon entering tumor cells, butyrate can induce the upregulation of CPT1A expression and activity, promoting the mitochondrial import of fatty acids and enhancing FAO, which in turn increases mitochondrial respiration and ATP production, thereby improving the metabolic adaptability of tumor cells to withstand immune or therapeutic stress. Conversely, knockdown of CPT1A disrupts this metabolic reprogramming pathway, reversing the butyrate-induced resistance effect and substantially restoring the antitumor efficacy of anti–PD-1 therapy (Zhu et al., 2025a). Acetyl-CoA carboxylase alpha (ACACA) is a key rate-limiting enzyme in fatty acid biosynthesis, catalyzing the carboxylation of acetyl-CoA to generate malonyl-CoA, thereby providing a direct substrate for FASN. Acetate primarily enhances the acetylation levels of H3K9, H3K27, and H3K56 at the promoter regions of ACACA and FASN, activating the expression of these lipogenic genes and promoting *de novo* lipid synthesis. Coupled with its role as a metabolic precursor for fatty acid synthesis, acetate functions as an epigenetic metabolite that supports cancer cell survival under hypoxic stress (Gao et al., 2016). IDO, a key enzyme in tryptophan metabolism, plays a central role in tumor immune regulation. Disruption of glutamine metabolism in MDSCs downregulates IDO expression in cancer cells, thereby perturbing the tryptophan metabolic pathway and ultimately suppressing cancer cell migration (Oh et al., 2020). In the colorectal cancer tumor microenvironment, *F. nucleatum* can inhibit AMPK activity by upregulating the expression of miR-130a-3p within tumor cells, thereby relieving its negative regulation on anabolic metabolism. This leads to activation of SREBF2 and its downstream cholesterol biosynthesis–related genes, promoting enhanced cholesterol biosynthesis in tumor cells, which in turn supports processes such as membrane synthesis, signal transduction, and cell proliferation and survival, ultimately driving tumor progression. Notably, butyrate can reverse this metabolic reprogramming effect by restoring AMPK activity or suppressing miR-130a-3p expression, thereby antagonizing the *F. nucleatum*–induced enhancement of cholesterol biosynthesis and its pro-tumorigenic effects (Sun et al., 2025).Secondly, regulating metabolism via signaling pathways constitutes another essential mechanism that warrants consideration. Researchers have explored the gut microbiota’s role in shaping the intratumoral microbes’ metabolic landscape. In a murine Lewis lung carcinoma model, oral administration of Akkermansia muciniphila—sourced endogenously or exogenously—together with MALDI-MSI–based spatial metabolomics, allowed *in situ* mapping of metabolite distributions within tumor tissues. The results demonstrated that *A. muciniphila* colonized the tumor site and markedly suppressed tumor growth and reprogrammed intratumoral energy and amino acid metabolism to exert its effects. Notably, it exhibited strong regulatory effects on critical metabolic pathways, including glutamine and lactate metabolism as well as pyrimidine biosynthesis, while modulating the expression of associated enzymes, thereby establishing a microbiota–metabolism interaction network that contributes to its antitumor activity (Zhu et al., 2023c) (**Figure 3**).

In summary, metabolic reprogramming in tumors mainly involves altering three key pathways: glucose, lipid, and amino acid metabolism. Microbial metabolites act as important external regulators, influencing tumor metabolism by two main mechanisms: controlling signaling pathways and directly affecting metabolic enzymes. However, current research is limited, mainly focusing on glucose metabolism, while studies on lipid and amino acid metabolism are comparatively few. The exact mechanisms behind these processes are still poorly understood, emphasizing the need for further detailed investigation.

## 4 Microbiota-derived metabolites in antitumor immunity and immunotherapy

### 4.1 Metabolic reprogramming of immune cells

Within the TME, tumor-associated immune cells undergo metabolic reprogramming as they compete with cancer cells for limited nutrients, engaging in continuous and dynamic reciprocal interactions (Reinfeld et al., 2021). In this context, microbial metabolites also play a critical role in remodeling the TME (Duan et al., 2025). Microbiota-derived metabolites participate in this process by modulating the metabolic state and function of immune cells, potentially attenuating or enhancing immune activity, thereby influencing tumor progression and responses to immunotherapy (**Figure 3**).

Among these immune populations, MDSCs exemplify how metabolic reprogramming enables immune cells to adapt to the nutrient-limited TME while exerting immunosuppressive functions. Tumor-infiltrating MDSCs exhibit markedly increased fatty acid uptake and oxygen consumption. This is primarily driven by the upregulation of key enzymes involved in fatty acid metabolism, including CPT1, acyl-CoA dehydrogenase, and 3-hydroxyacyl-CoA dehydrogenase, thereby facilitating FAO. Evidence also indicates that activation of G-CSF, GM-CSF, and the STAT3/STAT5 signaling pathways upregulates CD36 expression, thereby enhancing lipid uptake by MDSCs and modulating their immunosuppressive functions, which in turn indirectly affects CD8^+^ T cell–mediated antitumor responses (Al-Khami et al., 2017). Similarly, TAMs exhibit metabolic adaptations that shape their polarization and influence tumor immunity. TAMs play a pivotal role within the tumor microenvironment, and their polarization states—M1 or M2—directly influence tumor immune evasion and metastatic potential. M1 macrophages exhibit antitumor activity, whereas M2 macrophages generally display immunosuppressive characteristics, promoting tumor growth and metastasis. The conversion of M1 macrophages into the M2 phenotype weakens immune-mediated tumor attack and facilitates tumor progression and dissemination (Yang et al., 2025). Following gut microbiota dysbiosis, the proportion of M2-like macrophages in the tumor microenvironment is significantly increased, accompanied by a reduction in SCFA levels in both blood and tumor tissues. Mechanistic studies have demonstrated that SCFAs can promote M1 macrophage polarization by activating glycolytic pathways and receptor-mediated signaling in tumor-associated macrophages, thereby improving the immune microenvironment of gliomas and enhancing prognosis (Zhou et al., 2024). Inhibition of GS induces metabolic reprogramming in macrophages, characterized by decreased glutamine levels, succinate accumulation, and enhanced glucose uptake, thereby promoting M1 macrophage polarization and enhancing the antitumor function of macrophages (Palmieri et al., 2017). On the other hand, intracellular lipid accumulation or exogenous fatty acid supplementation can induce ROS production and promote the secretion of immunosuppressive cytokines. This process drives macrophages toward a pro-tumorigenic M2 phenotype, thereby enhancing the invasiveness and migratory capacity of cancer cells (Teng et al., 2023).Collectively, macrophage polarization is modulated by multiple metabolic factors within the microenvironment as well as microbial metabolites, representing a potential strategy for remodeling the tumor microenvironment. As different T cell subsets and differentiation states display distinct metabolic profiles (Wei et al., 2021). Memory T cells primarily rely on FAO and glucose-driven OXPHOS to maintain longevity and functional stability. In a lactate-enriched environment, regulatory Tregs rely on MCT1 to uptake lactate, which induces nuclear translocation of the NFAT, thereby upregulating PD-1 expression and enhancing their immunosuppressive function (Kumagai et al., 2022). In contrast, regulatory Tregs predominantly depend on glucose-driven OXPHOS as their primary energy source (O'Sullivan et al., 2018). Glutamine metabolism has been shown to support the proliferation and effector differentiation of Th1 and CD8^+^ T cells (Johnson et al., 2018). T cells in malignant ovarian cancer ascites display restricted glucose uptake accompanied by impaired N-linked glycosylation, suppressing the IRE1α/XBP1 signaling pathway, compromising mitochondrial function, and aberrant IFN-γ expression. Interventions targeting N-linked glycosylation, inhibiting the IRE1α/XBP1 pathway, or downregulating glutamine transporters can restore T cell glutamine uptake and metabolic efficiency, thereby promoting a shift from glycolysis toward glutamine-dependent metabolism (Song et al., 2018).

The discussion above primarily focuses on the intrinsic metabolic reprogramming of individual immune cell types; however, these metabolic programs do not operate in isolation. Various metabolites dynamically interact within the tumor microenvironment, forming cross-talk networks that further modulate immune cell functions. For example, lactate upregulates H3K18 lactylation (H3K18la), and the transcription factor CREB1 together with its coactivator EP300 directly binds to the B7-H3 promoter, leading to increased B7-H3 expression, which promotes tumor progression by reducing the proportion and cytotoxicity of tumor-infiltrating CD8^+^ T cells and enhances the efficacy of anti–PD-1 therapy (Ma et al., 2025), It can also enhance the immunosuppressive function of Treg cells by inducing lactylation of lysine 72 (Lys72) on MOESIN, which promotes its interaction with transforming growth factor β receptor I (TGF-βRI) (Gu et al., 2022). Furthermore, in IL-4–stimulated macrophages, exogenous lactate can substitute for glucose to sustain M2 polarization, suppress CD8^+^ T cell proliferation, and promote tumor progression *in vivo*, representing a lactate-mediated “metabolism–epigenetic” axis (Noe et al., 2021).Meanwhile, SCFAs can exert selective effects on different T cell subsets: butyrate promotes IFN-γ and T-bet expression and enhances inflammatory responses under Th1-polarizing conditions by inhibiting HDAC activity, thereby inducing epigenetic remodeling and metabolic reprogramming, whereas it suppresses IL-17 production under Th17-polarizing conditions (Chen et al., 2019),Propionate promotes Treg differentiation, thereby establishing a localized immunosuppressive environment (Arpaia et al., 2013).Similarly, IDO suppresses IL-6 production via the GCN2 pathway, thereby maintaining the suppressive phenotype of Foxp3^+^ Tregs; when IDO is absent or inhibited, IL-6 secreted by activated plasmacytoid dendritic cells (pDCs) promotes the conversion of Tregs into Th17-like cells, enhancing CD8^+^ T cell activity and antitumor immunity (Sharma et al., 2009).Overall, the function of immune cells within the tumor microenvironment is shaped not only by the intrinsic metabolic reprogramming of individual cell types but also by the cross-talk among various metabolites, forming a dynamic regulatory circuit that influences tumor immune evasion, progression, and response to immunotherapy, highlighting the tightly coupled relationship between tumor metabolism and immune regulation.

### 4.2 Impact of microbial metabolites on immunotherapy

Immunotherapy represents a major breakthrough in cancer treatment, exerting antitumor effects by alleviating immune suppression, enhancing T-cell function, and remodeling the tumor microenvironment. The underlying mechanisms include blocking immune checkpoints such as PD-1/PD-L1 and CTLA-4 to restore T-cell function (Topalian et al., 2012); enhancing tumor antigen recognition and cytotoxicity through adoptive cell therapies such as CAR-T (Simpson et al., 2013); modulating the immune microenvironment to increase CD8^+^ T-cell infiltration while reducing Treg and MDSC-mediated suppression (Deng et al., 2014); and activating antigen-specific T cells to establish immune memory, thereby strengthening defense against tumor recurrence or metastasis (Song et al., 2025).However, the clinical response rate to ICIs remains relatively low. The evaluation of immunotherapeutic efficacy typically relies on conventional immune biomarkers. PD-1/PD-L1 and CTLA-4 are classical immune checkpoint markers; the proportion and cytotoxicity of CD8^+^ T cells reflect effector activity, whereas the abundance of Tregs and MDSCs indicates the degree of immunosuppression. Additionally, cytokines such as IFN-γ, IL-10, and TNF-α can serve as indicators of immune activity (Davies et al., 2024). Recent studies have revealed that the antitumor immune activity of immune cells is modulated by the gut microbiota and its metabolites. Metabolomics offers a more refined approach to immune monitoring, enabling more accurate prediction of dynamic alterations in the tumor immune microenvironment and responses to immunotherapy.

#### 4.2.1 PD-1,PD-L1

In immunotherapy, the expression levels of PD-1 and its ligand PD-L1 serve as critical indicators for evaluating tumor immunosuppression and predicting the efficacy of ICIs.PD-1 is primarily expressed on the surface of activated T cells, B cells, and NK cells. When it binds to PD-L1 expressed on tumor cells or immunosuppressive cells, this interaction inhibits T cell proliferation, cytokine secretion, and cytotoxic activity, thereby contributing to the establishment of an immune evasion microenvironment. A prospective biomarker cohort study involving solid tumors found that higher concentrations of certain sSCFAs were significantly associated with longer progression (Nomura et al., 2020). In addition, metabolomic analysis of the gut microbiota in 11 patients with non-small cell lung cancer (NSCLC) receiving anti-PD-1 antibody therapy revealed that elevated levels of SCFAs, lysine, and niacin were significantly associated with long-term clinical benefit. These findings suggest that identifying microbiota-associated “metabolic biomarkers” may help distinguish early progressors from long-term responders, thereby improving the precision of immunotherapy (Botticelli et al., 2020). Mechanistically, serum levels of SCFAs were higher in NSCLC patients who responded to ICI therapy than in non-responders, and positively correlated with PD-1 expression on peripheral CD8^+^ T cells and Vγ9Vδ2 (Vδ2^+^) T cells. Butyrate increased H3K27ac enrichment at the promoter regions of *Pdcd1* and *Cd28* in human CD8^+^ T cells, thereby upregulating PD-1 and CD28 expression and enhancing the therapeutic efficacy of anti-PD-1 treatment. Under α-CD3/CD28 stimulation, butyrate-pretreated CD8^+^ T cells exhibited stronger TCR signaling activation, with increased phosphorylation of Lck, Zap70, LAT, and PLC-γ1, leading to greater CD8^+^ T cell infiltration into tumors, elevated production of IFN-γ and TNF-α, and enhanced sensitivity to TCR stimulation (Zhu et al., 2023b). Moreover, at specific concentrations, isobutyric acid significantly reduced tumor cell numbers in *in vitro* co-culture systems of cancer cells and T cells. It increased PD-1 expression on both CD4^+^ and CD8^+^ T cells, upregulated activation markers such as HLA-DR and ICOS, elevated *IFN-γ* mRNA levels, and decreased *FOXP3* mRNA expression. When combined with anti-PD-1 therapy, isobutyric acid enhanced antitumor efficacy, as evidenced by higher intratumoral expression of immune-related genes including *IFNG*, *ICOS*, and *PDCD1*. However, isobutyric acid did not alter PD-L1 or MHC I expression on tumor cells, nor did it significantly affect the levels of immunosuppressive cytokines such as IL-10 or TGF-β, suggesting that its potentiating effect primarily arises from direct modulation of T cells combined with its tumor-suppressive activity (Murayama et al., 2024). Upregulation of PD-1 expression on T cells by isobutyric acid may potentiate the efficacy of anti–PD-1 antibodies, while increased *IFN-γ* expression activates effector T cells and reduced *FOXP3* expression suppresses regulatory T cell function. In murine models, the combination of is butyric acid with anti–PD-1 antibody markedly inhibited tumor growth, with tumor volumes reduced by approximately 80% and 60% compared with control and anti–PD-1 monotherapy groups, respectively. This effect was accompanied by enhanced CD3^+^ T cell infiltration within tumor tissues and elevated expression of *IFN-γ*, *ICOS*, and *PDCD1*, indicating an augmented immune response. Moreover, the study excluded the involvement of immune escape mechanisms related to IL-10, TGF-β, or alterations in PD-L1/MHC I expression on tumor cells (Murayama et al., 2024).

In summary, microbial metabolites can modulate antitumor immunity by epigenetically regulating key immune checkpoints and enhancing T cell activation and effector functions, providing mechanistic insight into how the gut microbiota influences the therapeutic response to PD-1 blockade.

### 4.2 Chimeric antigen receptor T cell engineering

Chimeric antigen receptor T (CAR-T) cell engineering is an advanced form of adoptive cellular immunotherapy in which patient-derived or donor T cells are genetically modified to express chimeric receptors capable of specifically recognizing tumor-associated antigens, thereby enabling potent and targeted antitumor immune responses (Duan et al., 2025). CARs typically consist of an extracellular antigen-recognition domain, a transmembrane domain, and an intracellular signaling domain, enabling the activation of T-cell proliferation, cytokine secretion, and direct cytotoxicity to eliminate tumor cells and remodel the immune microenvironment. CAR-T therapy has achieved remarkable efficacy in B-cell acute lymphoblastic leukemia, certain lymphomas, and multiple myeloma; however, its application in solid tumors remains challenging due to tumor antigen heterogeneity, immunosuppressive tumor microenvironment, limited T-cell infiltration, and risks such as cytokine release syndrome (Althafar, 2025). Recent studies indicate that microbial metabolites can modulate the antitumor functions of CAR T cells.

For example, SCFAs can limit the anti-tumor activity of anti-CTLA-4 immunotherapy. Specifically, anti-CTLA-4 treatment typically upregulates the expression of co-stimulatory molecules CD80/CD86 and MHC II on dendritic cells, whereas butyrate inhibits these upregulations, as well as the expression of ICOS on T cells (a T cell activation marker) and the accumulation of tumor-specific and memory T cells, thereby dampening immune activity. SCFAs can suppress the immunostimulatory effects of anti-CTLA-4 by modulating dendritic cell function and T cell activation and memory formation (Coutzac et al., 2020). Simultaneously, Valerate can directly enter the metabolic network of CAR T cells, being converted into citrate via dual pathways through acetyl-CoA and succinyl-CoA, and subsequently generating nuclear acetyl-CoA under the catalysis of ACLY, thereby promoting the acetylation of histone H3K9/14 and H3K27. Concurrently, it inhibits HDAC1 activity, establishing a metabolically driven epigenetic activation effect. Valerate treatment enhances mTOR signaling, promotes mitochondrial biogenesis and oxidative metabolism, and boosts the energy supply and sustained functionality of CAR T cells. This metabolic-epigenetic reprogramming skews CAR T cells toward a naïve-like phenotype, reduces the proportion of exhausted populations, and significantly improves *in vivo* proliferation, tumor infiltration, and long-term antitumor efficacy, which has been validated in both patient cohorts and murine models (Staudt et al., 2025). Moreover, The use of broad-spectrum antibiotics was associated with poorer survival outcomes in patients with large B-cell lymphoma receiving anti-CD19 CAR-T therapy. In the discovery subcohort, these antibiotics induced significant dysregulation of gut microbiome function, accompanied by alterations in gut and blood metabolomes, including reductions in SCFAs, a finding that was recapitulated in an external validation cohort. In antibiotic-treated patients, levels of indole and TMAO were decreased, and these effects were confirmed in immune-competent CAR-T mouse models, where meropenem-induced microbiome dysbiosis led to systemic metabolic perturbations and reduced CAR-T efficacy. Furthermore, SCFAs were shown to enhance the metabolic fitness of CAR-T cells, thereby improving their tumor-killing capacity, suggesting that modulation of the microbiome may optimize CAR-T immunotherapy (Prasad et al., 2025).

Based on these mechanisms, researchers have proposed a “metabolite-guided CAR T cell engineering” strategy, and preclinical studies have shown that β-Hydroxybutyrate (BHB) derived from a ketogenic diet can significantly enhance the anti-tumor activity of CAR-T cells. Mechanistic studies indicate that CAR-T cells preferentially utilize BHB as a carbon source to boost the TCA cycle and oxidative phosphorylation, while BHB-derived acetyl-CoA enters the nucleus and acetylates histones, regulating the expression of key effector and memory genes. Clinically, serum BHB levels in CART19 patients positively correlate with CAR-T expansion, and supplementation of BHB during ex vivo manufacturing markedly enhances patient T cell proliferation. These findings have prompted the initiation of the first-in-human clinical trial investigating BHB supplementation during CART19 therapy for relapsed or refractory B cell lymphoma (Liu et al., 2024). In the future, integrating single-cell sequencing and metabolomics to elucidate the specific mechanisms by which different microbial metabolites influence CAR-T cell metabolic reprogramming will enable a systematic evaluation of their multi-level regulation of CAR-T function and the tumor microenvironment, thereby providing actionable metabolic intervention targets for personalized CAR-T cell engineering.

Finally, Most current studies are based on tumor-bearing mouse models, and their feasibility and therapeutic efficacy in humans remain to be clinically validated. Clinical investigations are required to systematically evaluate the optimal dosage, strain stability, and colonization capacity of the administered microbes in the gut, as well as to determine the appropriate intervention window, dosing frequency, and timing of combination with immune checkpoint inhibitors. In addition, indirect effects such as intestinal barrier restoration and inflammation alleviation may influence the tumor microenvironment, and these mechanisms warrant further elucidation. In patients, microbial metabolites are typically assessed by measuring their serum concentrations; however, their levels are influenced by factors such as intestinal production, absorption efficiency, tissue distribution, and metabolic clearance, leading to substantial interindividual variability. Moreover, differences in gut microbiota composition across tumor types or patient populations highlight the need for large-scale human studies to comprehensively evaluate these effects.

## 5 Limitations and future perspectives

**Figure 4:**
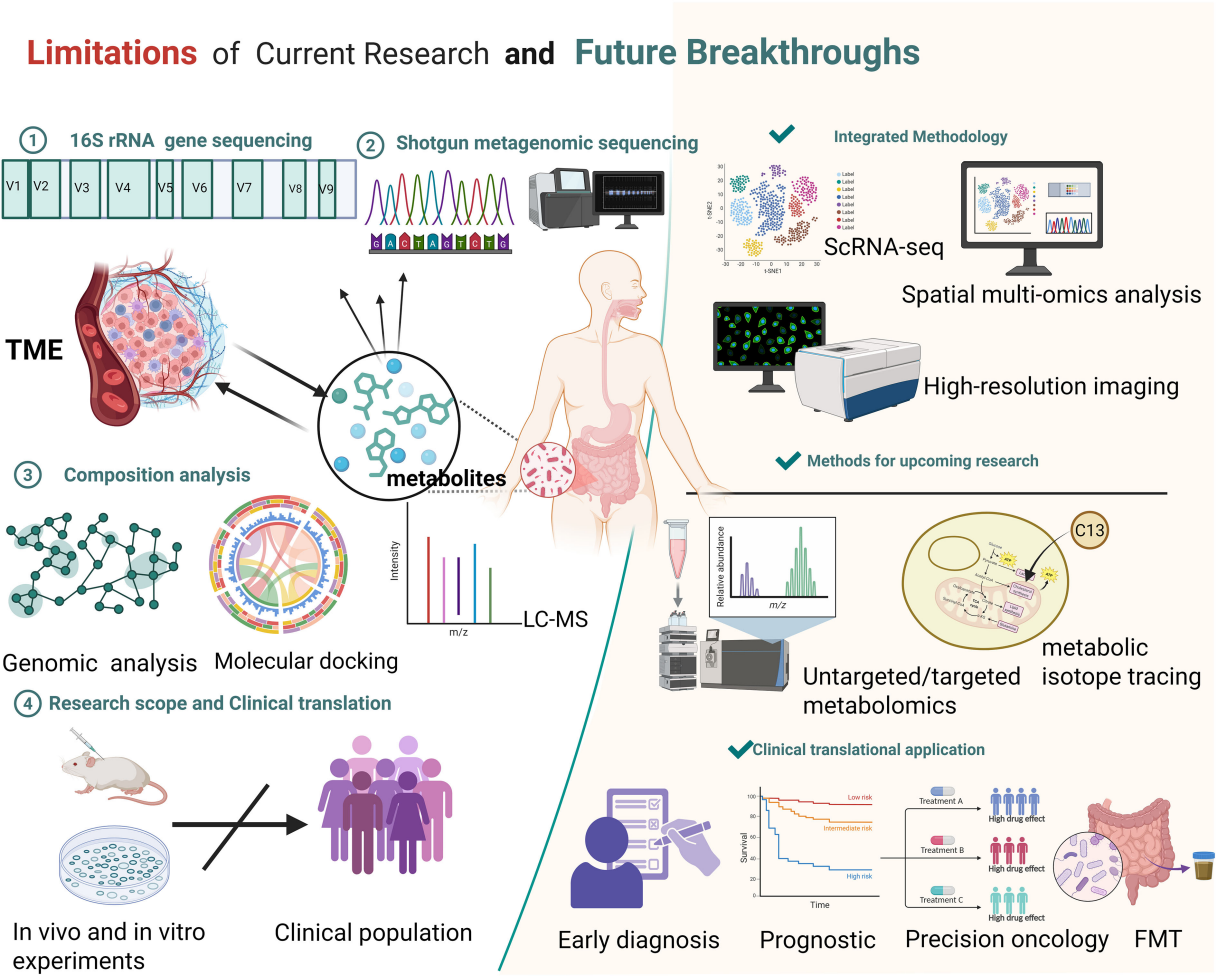
Summary of current research limitations on the microbiota and their metabolites, along with prospective avenues for future breakthroughs. Limitations: Current methods like 16S rRNA gene sequencing, shotgun metagenomic sequencing, composition analysis (genomic analysis, molecular docking, LC-MS), and research scope (mainly *in vivo*/*in vitro* experiments with limited clinical population translation) have constraints. Future Breakthroughs: Employ integrated methodologies (ScRNA-seq, spatial multi-omics analysis, high-resolution imaging), advanced research methods (untargeted/targeted metabolomics, metabolic isotope tracing), and promote clinical translational applications (early diagnosis, prognostic assessment, precision oncology, FMT). Created in https://BioRender.com.

Although significant progress has been made in the study of metabolites in recent years, numerous challenges and limitations remain. This review categorizes these issues into four domains: methodological approaches, functional mechanisms, research scope, and clinical translation, and discusses potential directions for future investigation (**Figure 4**).

From a methodological standpoint, microbial communities’ composition, abundance, and diversity are typically assessed using 16S rRNA sequencing and shotgun metagenomics in conjunction with public databases. Their functions can be inferred using tools such as PICRUSt and Tax4Fun (MatChado et al., 2024). However, genome-based functional inference and metabolic prediction indicate only potential capacities and cannot fully capture the active metabolic processes *in vivo*. Moreover, current metabolomic approaches remain limited in reliably determining the precise origin of specific metabolites under physiological conditions. In terms of functional mechanisms, Most studies concentrate on the specific microbial metabolites, overlooking potential synergistic or antagonistic interactions among metabolites and lacking a systematic investigative framework. Additionally, metabolites may exert contrasting effects depending on the biological context. Tumor heterogeneity can modulate the effects of microbial metabolites, with different tumor types potentially exhibiting distinct biological responses to the same metabolite. Even within a single tumor type, genetic alterations and epigenetic landscape may further influence these responses (Lei et al., 2025). These observations suggest that the role of microbial metabolites in tumors should be investigated in the context of multiple biological factors, rather than being simplistically classified as tumor-promoting or tumor-suppressing. In terms of research scope and clinical translation, current studies have primarily focused on *in vitro* experiments and animal models, with a lack of large-scale cohort studies and prospective clinical trials to systematically evaluate the clinical value of microbial metabolites in prognostic assessment and prediction of therapeutic responses. Moreover, similar metabolic alterations can be induced by multiple diseases, potentially compromising the specificity and accuracy of these metabolites in clinical diagnosis. Therefore, despite their potential as biomarkers, microbial metabolites still face significant challenges in direct clinical application.

To overcome these limitations, researchers are actively exploring corresponding solutions while also providing guidance for future investigations. First, integrating multiple innovative technologies with metabolomics enables a more comprehensive characterization of tumor metabolic features. For instance, isotope tracing facilitates the determination of metabolic activity across different organs, and the combination of isotope labeling with intratumoral spatial metabolomics in patient biopsies reveals the spatial heterogeneity of metabolic processes (Wang et al., 2022). Simultaneously, the integration of isotope tracing with spatial single-cell technologies enables high-resolution, cell-specific mapping of metabolic activities within tumor tissues, allowing dynamic monitoring of intracellular metabolic processes and their spatial distribution (Buglakova et al., 2024). Second, expanding the research scope by integrating microbiome data with host genetic and epigenetic information allows for a deeper understanding of individualized variations and functional relationships in host–microbiota–metabolite interactions (Garcia-Etxebarria et al., 2021; Liang et al., 2024). Finally, to enhance the clinical translatability of these findings, systematic evaluation within clinical intervention strategies is essential, employing multiple therapeutic approaches to advance more precise and personalized cancer treatment. For example, dietary interventions in melanoma patients have been shown to improve the efficacy of ICB therapy (Spencer et al., 2021); The engineered *Escherichia coli* Nissle 1917 strain can deliver 5-aminolevulinic acid (5-ALA) to colorectal cancer cells, thereby inducing selective cytotoxicity (Chen et al., 2021); Additionally, FMT combined with ICIs has been shown to enhance antitumor immune responses (Elkrief and Routy, 2021), developing metabolite-centered therapeutic strategies holds significant promise. (**Table 2**) Moreover, metabolite-based biomarkers demonstrate potential for patient stratification, dynamic monitoring of treatment responses, and prognosis prediction, thereby providing valuable support for clinical decision-making (Zhai et al., 2015).

**Table 2:** Clinical trials of microbiota-based interventions combined with immune checkpoint inhibitors.

Type	ID	Patient	Phase	ICIs	Number
FMT	NCT03341143	MM	II	pembrolizumab +Nivolumab	15
FMT	NCT04758507	mCRC	II	Pembrolizumab+ Axitinib	37
FMT	NCT04729322	mCRC+SBA	II	Pembrolizumab or Nivolumab	40
FMT	NCT04521075	NSCLC+MM	II	Pembrolizumab or Nivolumab	40
FMT	NCT04116775	MM	I	Pembrolizumab or Nivolumab	40
FMT	NCT05279677	mCRC	II	Sintilimab	40
FMT	NCT04521075	NSCLC+MM	I	Nivolumab	
FMT	NCT04130763	GI	I	Pembrolizumab or Nivolumab	10
FMT	NCT04924374	NSCLC	II	Pembrolizumab or Nivolumab	20
FMT	NCT03772899	MM	I	Nivolumab	20
FMT	NCT03353402	MM	I	Pembrolizumab	10
FMT	NCT03341143	MM	II	Pembrolizumab	20
GEN-001	NCT05998447	BTC	I	Pembrolizumab	56
CBM588	NCT03829111	mRCC	I	Nivolumab+Ipilimumab	30
VE800	NCT04208958	MM+CRC	I	Nivolumab	111
MET4	NCT03686202	ST	III	Ipilimumab	20
SYNB1891	NCT04167137	LYM	I	Atezolizumab	20
Dietary Intervention	NCT06391099	MM	I	Nivolumab+Lpilimumab	Not reported
Probiotics and Prebiotics	NCT06250335	MM	II	Lpilimumab+Nivolumab	Not reported
Nanomedicine-based Drug Delivery	NCT03589339	HNSCC	I	Nivolumab	60
CAR-T	NCT00902044	Sarcoma	I	HER2-CAR T	36
CAR-T	NCT04483778	Solid tumor	I	B7-H3-CAR T	68
CAR-T	NCT05168423	GBM	I	CART-EGFR-IL13Ra2	18

In summary, microbial metabolites, as a promising new class of tumor therapeutic targets, not only provide a novel perspective for elucidating the mechanisms of tumorigenesis but also lay the foundation for the development of individualized and precision therapeutic strategies. Although their clinical translation still faces numerous challenges, these metabolites hold significant research value and broad potential for future cancer treatment.

## References

[B1] Al-Khami A. A. Zheng L. Del Valle L. Hossain F. Wyczechowska D. Zabaleta J. . (2017). Exogenous lipid uptake induces metabolic and functional reprogramming of tumor-associated myeloid-derived suppressor cells. Oncoimmunology 6, e1344804. doi: 10.1080/2162402x.2017.1344804, PMID: 29123954 PMC5665069

[B2] Althafar Z. M. (2025). Engineered immune cell therapies for solid tumors: pharmacological advances, clinical outcomes, and future directions. Front. Pharmacol. 16. doi: 10.3389/fphar.2025.1614325, PMID: 40575784 PMC12198184

[B3] Anastasaki C. Mu R. Kernan C. M. Li X. Barakat R. Koleske J. P. . (2025). Aberrant coupling of glutamate and tyrosine kinase receptors enables neuronal control of brain-tumor growth. Neuron. S0896-6273 (25), 00591-4. doi: 10.1016/j.neuron.2025.08.005, PMID: 40897174 PMC12416319

[B4] Arpaia N. Campbell C. Fan X. Dikiy S. van der Veeken J. deRoos P. . (2013). Metabolites produced by commensal bacteria promote peripheral regulatory T-cell generation. Nature 504, 451–455. doi: 10.1038/nature12726, PMID: 24226773 PMC3869884

[B5] Baxter N. T. Schmidt A. W. Venkataraman A. Kim K. S. Waldron C. Schmidt T. M. (2019). Dynamics of human gut microbiota and short-chain fatty acids in response to dietary interventions with three fermentable fibers. mBio 10, e02566-18. doi: 10.1128/mBio.02566-18, PMID: 30696735 PMC6355990

[B6] Beg M. Abdullah N. Thowfeik F. S. Altorki N. K. McGraw T. E. (2017). Distinct Akt phosphorylation states are required for insulin regulated Glut4 and Glut1-mediated glucose uptake. Elife 6, e26896. doi: 10.7554/eLife.26896, PMID: 28589878 PMC5462539

[B7] Botticelli A. Vernocchi P. Marini F. Quagliariello A. Cerbelli B. Reddel S. . (2020). Gut metabolomics profiling of non-small cell lung cancer (NSCLC) patients under immunotherapy treatment. J. Transl. Med. 18, 49. doi: 10.1186/s12967-020-02231-0, PMID: 32014010 PMC6998840

[B8] Buglakova E. Ekelöf M. Schwaiger-Haber M. Schlicker L. Molenaar M. R. Shahraz M. . (2024). Spatial single-cell isotope tracing reveals heterogeneity of *de novo* fatty acid synthesis in cancer. Nat. Metab. 6, 1695–1711. doi: 10.1038/s42255-024-01118-4, PMID: 39251875 PMC11422168

[B9] Cao M. Zhang Z. Han S. Lu X. (2019). Butyrate inhibits the proliferation and induces the apoptosis of colorectal cancer HCT116 cells *via* the deactivation of mTOR/S6K1 signaling mediated partly by SIRT1 downregulation. Mol. Med. Rep. 19, 3941–3947. doi: 10.3892/mmr.2019.10002, PMID: 30864709

[B10] Che Y. Chen G. Guo Q. Duan Y. Feng H. Xia Q. (2023). Gut microbial metabolite butyrate improves anticancer therapy by regulating intracellular calcium homeostasis. Hepatology 78, 88–102. doi: 10.1097/hep.0000000000000047, PMID: 36947402

[B11] Chen J. Li X. Liu Y. Su T. Lin C. Shao L. . (2021). Engineering a probiotic strain of Escherichia coli to induce the regression of colorectal cancer through production of 5-aminolevulinic acid. Microb. Biotechnol. 14, 2130–2139. doi: 10.1111/1751-7915.13894, PMID: 34272828 PMC8449674

[B12] Chen J. Ye Z. Huang C. Qiu M. Song D. Li Y. . (2022a). Lipid nanoparticle-mediated lymph node-targeting delivery of mRNA cancer vaccine elicits robust CD8(+) T cell response. Proc. Natl. Acad. Sci. U.S.A. 119, e2207841119. doi: 10.1073/pnas.2207841119, PMID: 35969778 PMC9407666

[B13] Chen L. Sun M. Wu W. Yang W. Huang X. Xiao Y. . (2019). Microbiota metabolite butyrate differentially regulates th1 and th17 cells' Differentiation and function in induction of colitis. Inflammation Bowel Dis. 25, 1450–1461. doi: 10.1093/ibd/izz046, PMID: 30918945 PMC6701512

[B14] Chen W. Wen L. Bao Y. Tang Z. Zhao J. Zhang X. . (2022b). Gut flora disequilibrium promotes the initiation of liver cancer by modulating tryptophan metabolism and up-regulating SREBP2. Proc. Natl. Acad. Sci. U.S.A. 119, e2203894119. doi: 10.1073/pnas.2203894119, PMID: 36534812 PMC9907126

[B15] Chen Y. Zhou Y. Ren R. Chen Y. Lei J. Li Y. (2024). Harnessing lipid metabolism modulation for improved immunotherapy outcomes in lung adenocarcinoma. J. Immunother. Cancer 12, e008811. doi: 10.1136/jitc-2024-008811, PMID: 38977328 PMC11256034

[B16] Ciscato F. Ferrone L. Masgras I. Laquatra C. Rasola A. (2021). Hexokinase 2 in cancer: A prima donna playing multiple characters. Int. J. Mol. Sci. 22, 4716. doi: 10.3390/ijms22094716, PMID: 33946854 PMC8125560

[B17] Consortium H. M. P. (2012). Structure, function and diversity of the healthy human microbiome. Nature 486, 207–214. doi: 10.1038/nature11234, PMID: 22699609 PMC3564958

[B18] Coutzac C. Jouniaux J. M. Paci A. Schmidt J. Mallardo D. Seck A. . (2020). Systemic short chain fatty acids limit antitumor effect of CTLA-4 blockade in hosts with cancer. Nat. Commun. 11, 2168. doi: 10.1038/s41467-020-16079-x, PMID: 32358520 PMC7195489

[B19] Cui X. Cui Y. Du T. Jiang X. Song C. Zhang S. . (2022). SHMT2 drives the progression of colorectal cancer by regulating UHRF1 expression. Can. J. Gastroenterol. Hepatol. 2022, 3758697. doi: 10.1155/2022/3758697, PMID: 35211429 PMC8863481

[B20] Davies D. Kamdar S. Woolf R. Zlatareva I. Iannitto M. L. Morton C. . (2024). PD-1 defines a distinct, functional, tissue-adapted state in Vδ1(+) T cells with implications for cancer immunotherapy. Nat. Cancer 5, 420–432. doi: 10.1038/s43018-023-00690-0, PMID: 38172341 PMC10965442

[B21] Deng L. Liang H. Burnette B. Beckett M. Darga T. Weichselbaum R. R. . (2014). Irradiation and anti-PD-L1 treatment synergistically promote antitumor immunity in mice. J. Clin. Invest. 124, 687–695. doi: 10.1172/jci67313, PMID: 24382348 PMC3904601

[B22] Dietrich C. Bagatolli L. A. Volovyk Z. N. Thompson N. L. Levi M. Jacobson K. . (2001). Lipid rafts reconstituted in model membranes. Biophys. J. 80, 1417–1428. doi: 10.1016/s0006-3495(01)76114-0, PMID: 11222302 PMC1301333

[B23] Donohoe D. R. Garge N. Zhang X. Sun W. O'Connell T. M. Bunger M. K. . (2011). The microbiome and butyrate regulate energy metabolism and autophagy in the mammalian colon. Cell Metab. 13, 517–526. doi: 10.1016/j.cmet.2011.02.018, PMID: 21531334 PMC3099420

[B24] Duan H. Xu B. Luo P. Chen T. Zou J. (2025). Microbial metabolites and their influence on the tumor microenvironment. Front. Immunol. 16. doi: 10.3389/fimmu.2025.1675677, PMID: 41050671 PMC12488609

[B25] Duncan S. H. Barcenilla A. Stewart C. S. Pryde S. E. Flint H. J. (2002). Acetate utilization and butyryl coenzyme A (CoA):acetate-CoA transferase in butyrate-producing bacteria from the human large intestine. Appl. Environ. Microbiol. 68, 5186–5190. doi: 10.1128/aem.68.10.5186-5190.2002, PMID: 12324374 PMC126392

[B26] Dupraz L. Magniez A. Rolhion N. Richard M. L. Da Costa G. Touch S. . (2021). Gut microbiota-derived short-chain fatty acids regulate IL-17 production by mouse and human intestinal γδ T cells. Cell Rep. 36, 109332. doi: 10.1016/j.celrep.2021.109332, PMID: 34233192

[B27] Elkrief A. Routy B. (2021). First clinical proof-of-concept that FMT can overcome resistance to ICIs. Nat. Rev. Clin. Oncol. 18, 325–326. doi: 10.1038/s41571-021-00502-3, PMID: 33742164

[B28] Furusawa Y. Obata Y. Fukuda S. Endo T. A. Nakato G. Takahashi D. . (2013). Commensal microbe-derived butyrate induces the differentiation of colonic regulatory T cells. Nature 504, 446–450. doi: 10.1038/nature12721, PMID: 24226770

[B29] Gao X. Lin S. H. Ren F. Li J. T. Chen J. J. Yao C. B. . (2016). Acetate functions as an epigenetic metabolite to promote lipid synthesis under hypoxia. Nat. Commun. 7, 11960. doi: 10.1038/ncomms11960, PMID: 27357947 PMC4931325

[B30] Gao Z. Yin J. Zhang J. Ward R. E. Martin R. J. Lefevre M. . (2009). Butyrate improves insulin sensitivity and increases energy expenditure in mice. Diabetes 58, 1509–1517. doi: 10.2337/db08-1637, PMID: 19366864 PMC2699871

[B31] Garcia-Etxebarria K. Clos-Garcia M. Telleria O. Nafría B. Alonso C. Iruarrizaga-Lejarreta M. . (2021). Interplay between genome, metabolome and microbiome in colorectal cancer. Cancers (Basel) 13, 6216. doi: 10.3390/cancers13246216, PMID: 34944836 PMC8699218

[B32] Geng H. W. Yin F. Y. Zhang Z. F. Gong X. Yang Y. (2021). Butyrate suppresses glucose metabolism of colorectal cancer cells *via* GPR109a-AKT signaling pathway and enhances chemotherapy. Front. Mol. Biosci. 8. doi: 10.3389/fmolb.2021.634874, PMID: 33855046 PMC8039130

[B33] Gkiouli M. Biechl P. Eisenreich W. Otto A. M. (2019). Diverse roads taken by (13)C-glucose-derived metabolites in breast cancer cells exposed to limiting glucose and glutamine conditions. Cells 8, 1113. doi: 10.3390/cells8101113, PMID: 31547005 PMC6829299

[B34] Goffredo M. Mass K. Parks E. J. Wagner D. A. McClure E. A. Graf J. . (2016). Role of gut microbiota and short chain fatty acids in modulating energy harvest and fat partitioning in youth. J. Clin. Endocrinol. Metab. 101, 4367–4376. doi: 10.1210/jc.2016-1797, PMID: 27648960 PMC5095239

[B35] Gong J. Lin Y. Zhang H. Liu C. Cheng Z. Yang X. . (2020). Reprogramming of lipid metabolism in cancer-associated fibroblasts potentiates migration of colorectal cancer cells. Cell Death Dis. 11, 267. doi: 10.1038/s41419-020-2434-z, PMID: 32327627 PMC7181758

[B36] Gong L. Luo J. Zhang Y. Yang Y. Li S. Fang X. . (2023). Nasopharyngeal carcinoma cells promote regulatory T cell development and suppressive activity *via* CD70-CD27 interaction. Nat. Commun. 14, 1912. doi: 10.1038/s41467-023-37614-6, PMID: 37024479 PMC10079957

[B37] Grubelnik V. Zmazek J. Marhl M. (2025). The synergistic impact of glycolysis, mitochondrial oxPhos, and PEP cycling on ATP production in beta cells. Int. J. Mol. Sci. 26, 1454. doi: 10.3390/ijms26041454, PMID: 40003920 PMC11855156

[B38] Gu J. Zhou J. Chen Q. Xu X. Gao J. Li X. . (2022). Tumor metabolite lactate promotes tumorigenesis by modulating MOESIN lactylation and enhancing TGF-β signaling in regulatory T cells. Cell Rep. 39, 110986. doi: 10.1016/j.celrep.2022.110986, PMID: 35732125

[B39] Hanahan D. Weinberg R. A. (2011). Hallmarks of cancer: the next generation. Cell 144, 646–674. doi: 10.1016/j.cell.2011.02.013, PMID: 21376230

[B40] Hinnebusch B. F. Meng S. Wu J. T. Archer S. Y. Hodin R. A. (2002). The effects of short-chain fatty acids on human colon cancer cell phenotype are associated with histone hyperacetylation. J. Nutr. 132, 1012–1017. doi: 10.1093/jn/132.5.1012, PMID: 11983830

[B41] Hou Y. Li J. Ying S. (2023). Tryptophan metabolism and gut microbiota: A novel regulatory axis integrating the microbiome, immunity, and cancer. Metabolites 13, 1166. doi: 10.3390/metabo13111166, PMID: 37999261 PMC10673612

[B42] Hsu H. P. Chu P. Y. Chang T. M. Huang K. W. Hung W. C. Jiang S. S. . (2023). Mitochondrial phosphoenolpyruvate carboxykinase promotes tumor growth in estrogen receptor-positive breast cancer *via* regulation of the mTOR pathway. Cancer Med. 12, 1588–1601. doi: 10.1002/cam4.4969, PMID: 35757841 PMC9883444

[B43] Ishiguro T. Ohata H. Sato A. Yamawaki K. Enomoto T. Okamoto K. (2017). Tumor-derived spheroids: Relevance to cancer stem cells and clinical applications. Cancer Sci. 108, 283–289. doi: 10.1111/cas.13155, PMID: 28064442 PMC5378268

[B44] Jiang M. Incarnato D. Modderman R. Lazaro A. A. Jonkers I. H. Bianchi F. . (2025). Low butyrate concentrations exert anti-inflammatory and high concentrations exert pro-inflammatory effects on macrophages. J. Nutr. Biochem. 144, 109962. doi: 10.1016/j.jnutbio.2025.109962, PMID: 40381959

[B45] Jin B. Miao Z. Pan J. Zhang Z. Yang Y. Zhou Y. . (2025). The emerging role of glycolysis and immune evasion in ovarian cancer. Cancer Cell Int. 25, 78. doi: 10.1186/s12935-025-03698-x, PMID: 40045411 PMC11881340

[B46] Johnson M. O. Wolf M. M. Madden M. Z. Andrejeva G. Sugiura A. Contreras D. C. . (2018). Distinct regulation of th17 and th1 cell differentiation by glutaminase-dependent metabolism. Cell 175, 1780–1795.e1719. doi: 10.1016/j.cell.2018.10.001, PMID: 30392958 PMC6361668

[B47] Kekuda R. Manoharan P. Baseler W. Sundaram U. (2013). Monocarboxylate 4 mediated butyrate transport in a rat intestinal epithelial cell line. Dig Dis. Sci. 58, 660–667. doi: 10.1007/s10620-012-2407-x, PMID: 23344966

[B48] Kim M. H. Kang S. G. Park J. H. Yanagisawa M. Kim C. H. (2013). Short-chain fatty acids activate GPR41 and GPR43 on intestinal epithelial cells to promote inflammatory responses in mice. Gastroenterology 145, 396–406.e391-310. doi: 10.1053/j.gastro.2013.04.056, PMID: 23665276

[B49] Kong S. C. Nøhr-Nielsen A. Zeeberg K. Reshkin S. J. Hoffmann E. K. Novak I. . (2016). Monocarboxylate transporters MCT1 and MCT4 regulate migration and invasion of pancreatic ductal adenocarcinoma cells. Pancreas 45, 1036–1047. doi: 10.1097/mpa.0000000000000571, PMID: 26765963

[B50] Kumagai S. Koyama S. Itahashi K. Tanegashima T. Lin Y. T. Togashi Y. . (2022). Lactic acid promotes PD-1 expression in regulatory T cells in highly glycolytic tumor microenvironments. Cancer Cell 40, 201–218.e209. doi: 10.1016/j.ccell.2022.01.001, PMID: 35090594

[B51] Labadie B. W. Bao R. Luke J. J. (2019). Reimagining IDO pathway inhibition in cancer immunotherapy *via* downstream focus on the tryptophan-kynurenine-aryl hydrocarbon axis. Clin. Cancer Res. 25, 1462–1471. doi: 10.1158/1078-0432.Ccr-18-2882, PMID: 30377198 PMC6397695

[B52] Lam K. C. Araya R. E. Huang A. Chen Q. Di Modica M. Rodrigues R. R. . (2021). Microbiota triggers STING-type I IFN-dependent monocyte reprogramming of the tumor microenvironment. Cell 184, 5338–5356.e5321. doi: 10.1016/j.cell.2021.09.019, PMID: 34624222 PMC8650838

[B53] Lei L. Hong L. L. Ling Z. N. Zhong Y. Hu X. Y. Li P. . (2021). A potential oncogenic role for PFKFB3 overexpression in gastric cancer progression. Clin. Transl. Gastroenterol. 12, e00377. doi: 10.14309/ctg.0000000000000377, PMID: 34193800 PMC8345915

[B54] Lei J. Wang X. Liu X. (2025). Microbiota-derived metabolites in the epigenetic regulation of the host. Sci. Bull. (Beijing). doi: 10.1016/j.scib.2025.09.030, PMID: 41047314

[B55] Li Q. Cao L. Tian Y. Zhang P. Ding C. Lu W. . (2018). Butyrate suppresses the proliferation of colorectal cancer cells *via* targeting pyruvate kinase M2 and metabolic reprogramming. Mol. Cell Proteomics 17, 1531–1545. doi: 10.1074/mcp.RA118.000752, PMID: 29739823 PMC6072541

[B56] Li Q. Ding C. Meng T. Lu W. Liu W. Hao H. . (2017). Butyrate suppresses motility of colorectal cancer cells *via* deactivating Akt/ERK signaling in histone deacetylase dependent manner. J. Pharmacol. Sci. 135, 148–155. doi: 10.1016/j.jphs.2017.11.004, PMID: 29233468

[B57] Li X. Jia Y. Li Y. Hei H. Zhang S. Qin J. (2025). Crosstalk between metabolic reprogramming and microbiota: implications for cancer progression and novel therapeutic opportunities. Front. Immunol. 16. doi: 10.3389/fimmu.2025.1582166, PMID: 40463381 PMC12129893

[B58] Li T. Tan Y. T. Chen Y. X. Zheng X. J. Wang W. Liao K. . (2023). Methionine deficiency facilitates antitumour immunity by altering m(6)A methylation of immune checkpoint transcripts. Gut 72, 501–511. doi: 10.1136/gutjnl-2022-326928, PMID: 35803704 PMC9933173

[B59] Liang L. Kong C. Li J. Liu G. Wei J. Wang G. . (2024). Distinct microbes, metabolites, and the host genome define the multi-omics profiles in right-sided and left-sided colon cancer. Microbiome 12, 274. doi: 10.1186/s40168-024-01987-7, PMID: 39731152 PMC11681701

[B60] Liu S. Guruprasad P. Han K. Paruzzo L. Shestov A. Kelly A. . (2024). Ketogenic diet enhances CAR T cell antitumor function *via* β-hydroxybutyrate. Blood 144(Supplement 1), 4–4. doi: 10.1182/blood-2024-208913

[B61] Liu L. Liu Y. Zhou X. He H. Chen N. Qin Y. . (2025). Sodium butyrate induces ferroptosis in colorectal cancer cells by promoting NCOA4-FTH1-mediated ferritinophagy. Int. Immunopharmacol 163, 115188. doi: 10.1016/j.intimp.2025.115188, PMID: 40652583

[B62] Luu M. Riester Z. Baldrich A. Reichardt N. Yuille S. Busetti A. . (2021). Microbial short-chain fatty acids modulate CD8(+) T cell responses and improve adoptive immunotherapy for cancer. Nat. Commun. 12, 4077. doi: 10.1038/s41467-021-24331-1, PMID: 34210970 PMC8249424

[B63] Ma Z. Yang J. Jia W. Li L. Li Y. Hu J. . (2025). Histone lactylation-driven B7-H3 expression promotes tumor immune evasion. Theranostics 15, 2338–2359. doi: 10.7150/thno.105947, PMID: 39990209 PMC11840737

[B64] Marxsen J. H. Stengel P. Doege K. Heikkinen P. Jokilehto T. Wagner T. . (2004). Hypoxia-inducible factor-1 (HIF-1) promotes its degradation by induction of HIF-alpha-prolyl-4-hydroxylases. Biochem. J. 381, 761–767. doi: 10.1042/bj20040620, PMID: 15104534 PMC1133886

[B65] MatChado M. S. Rühlemann M. Reitmeier S. Kacprowski T. Frost F. Haller D. . (2024). On the limits of 16S rRNA gene-based metagenome prediction and functional profiling. Microb. Genom 10, 001203. doi: 10.1099/mgen.0.001203, PMID: 38421266 PMC10926695

[B66] Mathew M. Nguyen N. T. Bhutia Y. D. Sivaprakasam S. Ganapathy V. (2024). Metabolic signature of warburg effect in cancer: an effective and obligatory interplay between nutrient transporters and catabolic/anabolic pathways to promote tumor growth. Cancers (Basel) 16, 504. doi: 10.3390/cancers16030504, PMID: 38339256 PMC10854907

[B67] Mirji G. Worth A. Bhat S. A. El Sayed M. Kannan T. Goldman A. R. . (2022). The microbiome-derived metabolite TMAO drives immune activation and boosts responses to immune checkpoint blockade in pancreatic cancer. Sci. Immunol. 7, eabn0704. doi: 10.1126/sciimmunol.abn0704, PMID: 36083892 PMC9925043

[B68] Mo Y. Lin L. Zhang J. Yu C. (2022). SOAT1 enhances lung cancer invasiveness by stimulating AKT-mediated mitochondrial fragmentation. Biochem. Cell Biol. 100, 68–74. doi: 10.1139/bcb-2021-0175, PMID: 34670102

[B69] Muir A. Danai L. V. Gui D. Y. Waingarten C. Y. Lewis C. A. Vander Heiden M. G. (2017). Environmental cystine drives glutamine anaplerosis and sensitizes cancer cells to glutaminase inhibition. Elife 6. doi: 10.7554/eLife.27713, PMID: 28826492 PMC5589418

[B70] Murayama M. Hosonuma M. Kuramasu A. Kobayashi S. Sasaki A. Baba Y. . (2024). Isobutyric acid enhances the anti-tumour effect of anti-PD-1 antibody. Sci. Rep. 14, 11325. doi: 10.1038/s41598-024-59677-1, PMID: 38760458 PMC11101641

[B71] Nedjadi T. Moran A. W. Al-Rammahi M. A. Shirazi-Beechey S. P. (2014). Characterization of butyrate transport across the luminal membranes of equine large intestine. Exp. Physiol. 99, 1335–1347. doi: 10.1113/expphysiol.2014.077982, PMID: 25172888

[B72] Noe J. T. Rendon B. E. Geller A. E. Conroy L. R. Morrissey S. M. Young L. E. A. . (2021). Lactate supports a metabolic-epigenetic link in macrophage polarization. Sci. Adv. 7, eabi8602. doi: 10.1126/sciadv.abi8602, PMID: 34767443 PMC8589316

[B73] Nomura M. Nagatomo R. Doi K. Shimizu J. Baba K. Saito T. . (2020). Association of short-chain fatty acids in the gut microbiome with clinical response to treatment with nivolumab or pembrolizumab in patients with solid cancer tumors. JAMA Netw. Open 3, e202895. doi: 10.1001/jamanetworkopen.2020.2895, PMID: 32297948 PMC7163404

[B74] O'Sullivan D. van der Windt G. J. W. Huang S. C. Curtis J. D. Chang C. H. Buck M. D. . (2018). Memory CD8(+) T cells use cell-intrinsic lipolysis to support the metabolic programming necessary for development. Immunity 49, 375–376. doi: 10.1016/j.immuni.2018.07.018, PMID: 30134202 PMC6167519

[B75] Oh M. H. Sun I. H. Zhao L. Leone R. D. Sun I. M. Xu W. . (2020). Targeting glutamine metabolism enhances tumor-specific immunity by modulating suppressive myeloid cells. J. Clin. Invest. 130, 3865–3884. doi: 10.1172/jci131859, PMID: 32324593 PMC7324212

[B76] Okumura S. Konishi Y. Narukawa M. Sugiura Y. Yoshimoto S. Arai Y. . (2021). Gut bacteria identified in colorectal cancer patients promote tumourigenesis *via* butyrate secretion. Nat. Commun. 12, 5674. doi: 10.1038/s41467-021-25965-x, PMID: 34584098 PMC8479117

[B77] Oliver A. Alkan Z. Stephensen C. B. Newman J. W. Kable M. E. Lemay D. G. (2024). Diet, microbiome, and inflammation predictors of fecal and plasma short-chain fatty acids in humans. J. Nutr. 154, 3298–3311. doi: 10.1016/j.tjnut.2024.08.012, PMID: 39173973 PMC11600052

[B78] Oncel S. Safratowich B. D. Lindlauf J. E. Liu Z. Palmer D. G. Briske-Anderson M. . (2024). Efficacy of butyrate to inhibit colonic cancer cell growth is cell type-specific and apoptosis-dependent. Nutrients 16, 529. doi: 10.3390/nu16040529, PMID: 38398853 PMC10892417

[B79] Palmieri E. M. Menga A. Martín-Pérez R. Quinto A. Riera-Domingo C. De Tullio G. . (2017). Pharmacologic or genetic targeting of glutamine synthetase skews macrophages toward an M1-like phenotype and inhibits tumor metastasis. Cell Rep. 20, 1654–1666. doi: 10.1016/j.celrep.2017.07.054, PMID: 28813676 PMC5575233

[B80] Pandit M. Kil Y. S. Ahn J. H. Pokhrel R. H. Gu Y. Mishra S. . (2023). Methionine consumption by cancer cells drives a progressive upregulation of PD-1 expression in CD4 T cells. Nat. Commun. 14, 2593. doi: 10.1038/s41467-023-38316-9, PMID: 37147330 PMC10162977

[B81] Pascual G. Majem B. Benitah S. A. (2024). Targeting lipid metabolism in cancer metastasis. Biochim. Biophys. Acta Rev. Cancer 1879, 189051. doi: 10.1016/j.bbcan.2023.189051, PMID: 38101461

[B82] Peng L. Li Z. R. Green R. S. Holzman I. R. Lin J. (2009). Butyrate enhances the intestinal barrier by facilitating tight junction assembly *via* activation of AMP-activated protein kinase in Caco-2 cell monolayers. J. Nutr. 139, 1619–1625. doi: 10.3945/jn.109.104638, PMID: 19625695 PMC2728689

[B83] Ping Y. Fan Q. Zhang Y. (2025). Modulating lipid metabolism improves tumor immunotherapy. J. Immunother. Cancer 13, e010824. doi: 10.1136/jitc-2024-010824, PMID: 39904563 PMC11795363

[B84] PrasadR. RehmanA. RehmanL. DarbaniyanF. BlumenbergV. SchubertM. L. . (2025). Antibiotic-induced loss of gut microbiome metabolic output correlates with clinical responses to CAR T-cell therapy. Blood 145, 823–839. doi: 10.1182/blood.2024025366, PMID: 39441941

[B85] Reichardt N. Duncan S. H. Young P. Belenguer A. McWilliam Leitch C. Scott K. P. . (2014). Phylogenetic distribution of three pathways for propionate production within the human gut microbiota. Isme J. 8, 1323–1335. doi: 10.1038/ismej.2014.14, PMID: 24553467 PMC4030238

[B86] Reid M. A. Allen A. E. Liu S. Liberti M. V. Liu P. Liu X. . (2018). Serine synthesis through PHGDH coordinates nucleotide levels by maintaining central carbon metabolism. Nat. Commun. 9, 5442. doi: 10.1038/s41467-018-07868-6, PMID: 30575741 PMC6303315

[B87] Reinfeld B. I. Madden M. Z. Wolf M. M. Chytil A. Bader J. E. Patterson A. R. . (2021). Cell-programmed nutrient partitioning in the tumour microenvironment. Nature 593, 282–288. doi: 10.1038/s41586-021-03442-1, PMID: 33828302 PMC8122068

[B88] Romano K. A. Vivas E. I. Amador-Noguez D. Rey F. E. (2015). Intestinal microbiota composition modulates choline bioavailability from diet and accumulation of the proatherogenic metabolite trimethylamine-N-oxide. mBio 6, e02481. doi: 10.1128/mBio.02481-14, PMID: 25784704 PMC4453578

[B89] Schütze A. Benndorf D. Püttker S. Kohrs F. Bettenbrock K. (2020). The Impact of ackA, pta, and ackA-pta Mutations on Growth, Gene Expression and Protein Acetylation in Escherichia coli K-12. Front. Microbiol. 11. doi: 10.3389/fmicb.2020.00233, PMID: 32153530 PMC7047895

[B90] Senthong V. Kiatchoosakun S. Wongvipaporn C. Phetcharaburanin J. Sritara P. Phrommintikul A. (2024). Trimethylamine-N-oxide and 5-year mortality: the role of gut microbiota-generated metabolite from the CORE-Thailand cohort. Sci. Rep. 14, 21264. doi: 10.1038/s41598-024-71479-z, PMID: 39261513 PMC11391081

[B91] Sharma M. D. Hou D. Y. Liu Y. Koni P. A. Metz R. Chandler P. . (2009). Indoleamine 2,3-dioxygenase controls conversion of Foxp3+ Tregs to TH17-like cells in tumor-draining lymph nodes. Blood 113, 6102–6111. doi: 10.1182/blood-2008-12-195354, PMID: 19366986 PMC2699232

[B92] Shen N. Wang Y. Sun X. Bai X. He J. Cui Q. . (2020). Expression of hypoxia-inducible factor 1α, glucose transporter 1, and hexokinase 2 in primary central nervous system lymphoma and the correlation with the biological behaviors. Brain Behav. 10, e01718. doi: 10.1002/brb3.1718, PMID: 32533646 PMC7428508

[B93] Shu X. Zhan P. P. Sun L. X. Yu L. Liu J. Sun L. C. . (2021). BCAT1 activates PI3K/AKT/mTOR pathway and contributes to the angiogenesis and tumorigenicity of gastric cancer. Front. Cell Dev. Biol. 9. doi: 10.3389/fcell.2021.659260, PMID: 34164393 PMC8215359

[B94] Simcox J. Lamming D. W. (2022). The central moTOR of metabolism. Dev. Cell 57, 691–706. doi: 10.1016/j.devcel.2022.02.024, PMID: 35316619 PMC9004513

[B95] Simpson T. R. Li F. Montalvo-Ortiz W. Sepulveda M. A. Bergerhoff K. Arce F. . (2013). Fc-dependent depletion of tumor-infiltrating regulatory T cells co-defines the efficacy of anti-CTLA-4 therapy against melanoma. J. Exp. Med. 210, 1695–1710. doi: 10.1084/jem.20130579, PMID: 23897981 PMC3754863

[B96] Song Y. Chen J. Zhang Y. Wu N. Zhu Y. Chen G. . (2025). Tumor-specific CXCR6 positive precursor CD8(+) T cells mediate tumor control in metastatic melanoma. Cell Oncol. (Dordr) 48, 693–708. doi: 10.1007/s13402-025-01040-1, PMID: 40192941 PMC12119687

[B97] Song M. Sandoval T. A. Chae C. S. Chopra S. Tan C. Rutkowski M. R. . (2018). IRE1α-XBP1 controls T cell function in ovarian cancer by regulating mitochondrial activity. Nature 562, 423–428. doi: 10.1038/s41586-018-0597-x, PMID: 30305738 PMC6237282

[B98] Spencer C. N. McQuade J. L. Gopalakrishnan V. McCulloch J. A. Vetizou M. Cogdill A. P. . (2021). Dietary fiber and probiotics influence the gut microbiome and melanoma immunotherapy response. Science 374, 1632–1640. doi: 10.1126/science.aaz7015, PMID: 34941392 PMC8970537

[B99] Staudt S. Nikolka F. Perl M. Franz J. Leblay N. Yuan X. K. . (2025). Metabolization of microbial postbiotic pentanoate drives anti-cancer CAR T cells. bioRxiv. doi: 10.1101/2024.08.19.608538, PMID: 39314273 PMC11418944

[B100] Sun Y. Lu J. Tung Lau E. Y. Zeng Y. Lam Li S. W. Au T. H. . (2025). Fusobacterium nucleatum enhances cholesterol biosynthesis in colorectal cancer *via* miR-130a-3p-mediated AMPK inhibition, a process counteracted by butyrate. Cancer Lett. 627, 217810. doi: 10.1016/j.canlet.2025.217810, PMID: 40414519

[B101] Tao T. Su Q. Xu S. Deng J. Zhou S. Zhuang Y. . (2019). Down-regulation of PKM2 decreases FASN expression in bladder cancer cells through AKT/mTOR/SREBP-1c axis. J. Cell Physiol. 234, 3088–3104. doi: 10.1002/jcp.27129, PMID: 30221356

[B102] Teng Y. Xu L. Li W. Liu P. Tian L. Liu M. (2023). Targeting reactive oxygen species and fat acid oxidation for the modulation of tumor-associated macrophages: a narrative review. Front. Immunol. 14. doi: 10.3389/fimmu.2023.1224443, PMID: 37545527 PMC10401428

[B103] Thangaraju M. Cresci G. A. Liu K. Ananth S. Gnanaprakasam J. P. Browning D. D. . (2009). GPR109A is a G-protein-coupled receptor for the bacterial fermentation product butyrate and functions as a tumor suppressor in colon. Cancer Res. 69, 2826–2832. doi: 10.1158/0008-5472.Can-08-4466, PMID: 19276343 PMC3747834

[B104] Tingstad R. H. Witczak O. Beajani S. Seo S. H. Løvsletten N. G. Skagen C. . (2025). Impact of short-chain fatty acids on glucose, fatty acid and leucine metabolism in primary human myotubes. Endocrinol. Diabetes Metab. 8, e70042. doi: 10.1002/edm2.70042, PMID: 40051343 PMC11885951

[B105] Topalian S. L. Hodi F. S. Brahmer J. R. Gettinger S. N. Smith D. C. McDermott D. F. . (2012). Safety, activity, and immune correlates of anti-PD-1 antibody in cancer. N Engl. J. Med. 366, 2443–2454. doi: 10.1056/NEJMoa1200690, PMID: 22658127 PMC3544539

[B106] Wang Y. Chen W. Wang Z. Cai S. Zhao X. Jin J. . (2025). Deciphering metabolic reprogramming of immune cells within the tumor microenvironment. J. Transl. Med. 23, 1055. doi: 10.1186/s12967-025-07069-y, PMID: 41053742 PMC12502548

[B107] Wang C. Shao L. Pan C. Ye J. Ding Z. Wu J. . (2019). Elevated level of mitochondrial reactive oxygen species *via* fatty acid β-oxidation in cancer stem cells promotes cancer metastasis by inducing epithelial-mesenchymal transition. Stem Cell Res. Ther. 10, 175. doi: 10.1186/s13287-019-1265-2, PMID: 31196164 PMC6567550

[B108] Wang S. van Geffen M. Venema K. Mommers A. Jonkers D. van Schooten F. J. . (2023b). Effect of protein fermentation products on gut health assessed in an *in vitro* model of human colon (TIM-2). Mol. Nutr. Food Res. 67, e2200574. doi: 10.1002/mnfr.202200574, PMID: 36808825

[B109] Wang W. Xiao Z. D. Li X. Aziz K. E. Gan B. Johnson R. L. . (2015). AMPK modulates Hippo pathway activity to regulate energy homeostasis. Nat. Cell Biol. 17, 490–499. doi: 10.1038/ncb3113, PMID: 25751139 PMC4380807

[B110] Wang L. Xing X. Zeng X. Jackson S. R. TeSlaa T. Al-Dalahmah O. . (2022). Spatially resolved isotope tracing reveals tissue metabolic activity. Nat. Methods 19, 223–230. doi: 10.1038/s41592-021-01378-y, PMID: 35132243 PMC10926149

[B111] Wang C. Yang Z. Xu E. Shen X. Wang X. Li Z. . (2021). Apolipoprotein C-II induces EMT to promote gastric cancer peritoneal metastasis *via* PI3K/AKT/mTOR pathway. Clin. Transl. Med. 11, e522. doi: 10.1002/ctm2.522, PMID: 34459127 PMC8351524

[B112] Wang Y. N. Zeng Z. L. Lu J. Wang Y. Liu Z. X. He M. M. . (2018). CPT1A-mediated fatty acid oxidation promotes colorectal cancer cell metastasis by inhibiting anoikis. Oncogene 37, 6025–6040. doi: 10.1038/s41388-018-0384-z, PMID: 29995871

[B113] Wang K. Zhang Y. Chen Z. N. (2023a). Metabolic interaction: tumor-derived lactate inhibiting CD8(+) T cell cytotoxicity in a novel route. Signal Transduct Target Ther. 8, 52. doi: 10.1038/s41392-023-01320-y, PMID: 36737430 PMC9898493

[B114] Wei J. Zheng W. Chapman N. M. Geiger T. L. Chi H. (2021). T cell metabolism in homeostasis and cancer immunity. Curr. Opin. Biotechnol. 68, 240–250. doi: 10.1016/j.copbio.2021.02.003, PMID: 33676144 PMC8137568

[B115] Weinhouse S. Wenner C. E. (1956). Metabolism of neoplastic tissue. IX. An isotope tracer study of glucose catabolism pathways in normal and neoplastic tissues. J. Biol. Chem. 222, 399–414., PMID: 13367012

[B116] Wen P. Wang G. Zhang N. Shao Q. Wang L. Qu F. . (2025). Frontiers and hot topics in tumor metabolic reprogramming: a bibliometric analysis from 2014 to 2023. Front. Oncol. 15. doi: 10.3389/fonc.2025.1570532, PMID: 40630197 PMC12234538

[B117] Wolfson R. L. Chantranupong L. Saxton R. A. Shen K. Scaria S. M. Cantor J. R. . (2016). Sestrin2 is a leucine sensor for the mTORC1 pathway. Science 351, 43–48. doi: 10.1126/science.aab2674, PMID: 26449471 PMC4698017

[B118] Wu Q. L. Fang X. T. Wan X. X. Ding Q. Y. Zhang Y. J. Ji L. . (2024). Fusobacterium nucleatum-induced imbalance in microbiome-derived butyric acid levels promotes the occurrence and development of colorectal cancer. World J. Gastroenterol. 30, 2018–2037. doi: 10.3748/wjg.v30.i14.2018, PMID: 38681125 PMC11045493

[B119] Xiu W. Chen Q. Wang Z. Wang J. Zhou Z. (2020). Microbiota-derived short chain fatty acid promotion of Amphiregulin expression by dendritic cells is regulated by GPR43 and Blimp-1. Biochem. Biophys. Res. Commun. 533, 282–288. doi: 10.1016/j.bbrc.2020.09.027, PMID: 32958255

[B120] Yamaguchi A. Mukai Y. Sakuma T. Narumi K. Furugen A. Yamada Y. . (2023). Monocarboxylate transporter 4 involves in energy metabolism and drug sensitivity in hypoxia. Sci. Rep. 13, 1501. doi: 10.1038/s41598-023-28558-4, PMID: 36707650 PMC9883486

[B121] Yang Y. Li S. To K. K. W. Zhu S. Wang F. Fu L. (2025). Tumor-associated macrophages remodel the suppressive tumor immune microenvironment and targeted therapy for immunotherapy. J. Exp. Clin. Cancer Res. 44, 145. doi: 10.1186/s13046-025-03377-9, PMID: 40380196 PMC12083052

[B122] Yu Q. Dai W. Ji J. Wu L. Feng J. Li J. . (2022). Sodium butyrate inhibits aerobic glycolysis of hepatocellular carcinoma cells *via* the c-myc/hexokinase 2 pathway. J. Cell Mol. Med. 26, 3031–3045. doi: 10.1111/jcmm.17322, PMID: 35429101 PMC9097842

[B123] Zhai L. Dey M. Lauing K. L. Gritsina G. Kaur R. Lukas R. V. . (2015). The kynurenine to tryptophan ratio as a prognostic tool for glioblastoma patients enrolling in immunotherapy. J. Clin. Neurosci. 22, 1964–1968. doi: 10.1016/j.jocn.2015.06.018, PMID: 26279502 PMC4548799

[B124] Zhang C. Liu J. Liang Y. Wu R. Zhao Y. Hong X. . (2013). Tumour-associated mutant p53 drives the Warburg effect. Nat. Commun. 4, 2935. doi: 10.1038/ncomms3935, PMID: 24343302 PMC3969270

[B125] Zhang C. Xu S. Yin C. Hu S. Liu P. (2025). The role of the mTOR pathway in breast cancer stem cells (BCSCs): mechanisms and therapeutic potentials. Stem Cell Res. Ther. 16, 156. doi: 10.1186/s13287-025-04218-4, PMID: 40158191 PMC11954216

[B126] Zhang Q. Zhao Q. Li T. Lu L. Wang F. Zhang H. . (2023). Lactobacillus plantarum-derived indole-3-lactic acid ameliorates colorectal tumorigenesis *via* epigenetic regulation of CD8(+) T cell immunity. Cell Metab. 35, 943–960.e949. doi: 10.1016/j.cmet.2023.04.015, PMID: 37192617

[B127] Zhang M. Zhou Q. Dorfman R. G. Huang X. Fan T. Zhang H. . (2016). Butyrate inhibits interleukin-17 and generates Tregs to ameliorate colorectal colitis in rats. BMC Gastroenterol. 16, 84. doi: 10.1186/s12876-016-0500-x, PMID: 27473867 PMC4967301

[B128] Zheng M. Yang X. Wu Q. Gong Y. Pang N. Ge X. . (2023). Butyrate attenuates hepatic steatosis induced by a high-fat and fiber-deficient diet *via* the hepatic GPR41/43-caMKII/HDAC1-CREB pathway. Mol. Nutr. Food Res. 67, e2200597. doi: 10.1002/mnfr.202200597, PMID: 36382553 PMC10078002

[B129] Zhou M. Wu J. Shao Y. Zhang J. Zheng R. Shi Q. . (2024). Short-chain fatty acids reverses gut microbiota dysbiosis-promoted progression of glioblastoma by up-regulating M1 polarization in the tumor microenvironment. Int. Immunopharmacol 141, 112881. doi: 10.1016/j.intimp.2024.112881, PMID: 39159556

[B130] Zhu Z. Cai J. Hou W. Xu K. Wu X. Song Y. . (2023c). Microbiome and spatially resolved metabolomics analysis reveal the anticancer role of gut Akkermansia muciniphila by crosstalk with intratumoral microbiota and reprogramming tumoral metabolism in mice. Gut Microbes 15, 2166700. doi: 10.1080/19490976.2023.2166700, PMID: 36740846 PMC9904296

[B131] Zhu R. Gu S. Tao Y. Zhang Y. (2025a). Butyrate confers colorectal cancer cell resistance to anti-PD-1 therapy by promoting CPT1A-mediated fatty acid oxidation. Discov. Oncol. 16, 935. doi: 10.1007/s12672-025-02686-x, PMID: 40423770 PMC12116955

[B132] Zhu Z. Huang J. Li X. Xing J. Chen Q. Liu R. . (2020). Gut microbiota regulate tumor metastasis *via* circRNA/miRNA networks. Gut Microbes 12, 1788891. doi: 10.1080/19490976.2020.1788891, PMID: 32686598 PMC7524358

[B133] Zhu X. Li K. Liu G. Wu R. Zhang Y. Wang S. . (2023b). Microbial metabolite butyrate promotes anti-PD-1 antitumor efficacy by modulating T cell receptor signaling of cytotoxic CD8 T cell. Gut Microbes 15, 2249143. doi: 10.1080/19490976.2023.2249143, PMID: 37635362 PMC10464552

[B134] Zhu Y. Li T. Ramos da Silva S. Lee J. J. Lu C. Eoh H. . (2017). A critical role of glutamine and asparagine γ-nitrogen in nucleotide biosynthesis in cancer cells hijacked by an oncogenic virus. mBio 8, e01179-17. doi: 10.1128/mBio.01179-17, PMID: 28811348 PMC5559638

[B135] Zhu G. Q. Tang Z. Huang R. Qu W. F. Fang Y. Yang R. . (2023a). CD36(+) cancer-associated fibroblasts provide immunosuppressive microenvironment for hepatocellular carcinoma *via* secretion of macrophage migration inhibitory factor. Cell Discov. 9, 25. doi: 10.1038/s41421-023-00529-z, PMID: 36878933 PMC9988869

[B136] Zhu Y. Zhou Z. Du X. Lin X. Liang Z. M. Chen S. . (2025b). Cancer cell-derived arginine fuels polyamine biosynthesis in tumor-associated macrophages to promote immune evasion. Cancer Cell 43, 1045–1060.e1047. doi: 10.1016/j.ccell.2025.03.015, PMID: 40185095

